# Silence as a way of niche adaptation: *mec*C-MRSA with variations in the accessory gene regulator (*agr*) functionality express kaleidoscopic phenotypes

**DOI:** 10.1038/s41598-020-71640-4

**Published:** 2020-09-08

**Authors:** Charlotte Huber, Ivonne Stamm, Wilma Ziebuhr, Gabriella Marincola, Markus Bischoff, Birgit Strommenger, Greta Jaschkowitz, Tessa Marciniak, Christiane Cuny, Wolfgang Witte, Joerg Doellinger, Christoph Schaudinn, Andrea Thürmer, Lennard Epping, Torsten Semmler, Antina Lübke-Becker, Lothar H. Wieler, Birgit Walther

**Affiliations:** 1 Advanced Light and Electron Microscopy (ZBS4), Robert Koch Institute, Berlin, Germany; 2 IDEXX GmbH, Ludwigsburg, Germany; 3 Institute for Molecular Infection Biology, University of Würzburg, Würzburg, Germany; 4 Institute of Medical Microbiology and Hygiene, Saarland University, Homburg, Germany; 5 National Reference Centre for Staphylococci and Enterococci, Robert Koch Institute, Wernigerode, Germany; 6 Proteomics and Spectroscopy (ZBS6), Robert Koch Institute, Berlin, Germany; 7 Genome Sequencing (MF2), Robert Koch Institute, Berlin, Germany; 8 Microbial Genomics (NG1), Robert Koch Institute, Berlin, Germany; 9 Institute of Microbiology and Epizootics, Freie Universität Berlin, Berlin, Germany; 10 Methodology and Research Infrastructure, Robert Koch Institute, Berlin, Germany

**Keywords:** Antimicrobials, Bacteria, Bacteriology, Biofilms, Microbial genetics, Microbiology

## Abstract

Functionality of the accessory gene regulator (*agr*) quorum sensing system is an important factor promoting either acute or chronic infections by the notorious opportunistic human and veterinary pathogen *Staphylococcus aureus*. Spontaneous alterations of the *agr* system are known to frequently occur in human healthcare-associated *S. aureus* lineages. However, data on *agr* integrity and function are sparse regarding other major clonal lineages. Here we report on the *agr* system functionality and activity level in *mec*C-carrying methicillin resistant *S. aureus* (MRSA) of various animal origins (n = 33) obtained in Europe as well as in closely related human isolates (n = 12). Whole genome analysis assigned all isolates to four clonal complexes (CC) with distinct *agr* types (CC599 *agr* I, CC49 *agr *II, CC130 *agr* III and CC1943 *agr* IV). Agr functionality was assessed by a combination of phenotypic assays and proteome analysis. In each CC, isolates with varying *agr* activity levels were detected, including the presence of completely non-functional variants. Genomic comparison of the *agr *I–IV encoding regions associated these phenotypic differences with variations in the *agr*A and *agr*C genes. The genomic changes were detected independently in divergent lineages, suggesting that *agr* variation might foster viability and adaptation of emerging MRSA lineages to distinct ecological niches.

## Introduction

Methicillin-resistant *Staphylococcus aureus* (MRSA) is among the leading causes of opportunistic infectious diseases in human and veterinary medicine worldwide^1,2^. Mobile genetic elements (MGEs) originally denominated as staphylococcal cassette chromosome *mec* (SCC*mec*) cause horizontal spread of methicillin resistance among staphylococci^3^, with either *mec*A or *mec*C^4,5^ as the resistance-mediating gene. So far, thirteen distinct SCC*mec* elements have been described, including the *mec*C-harbouring element SCC*mec* XI^5,6^. When these MRSA were first isolated from specimens of human and ruminant hosts back in 2011^7–9^, detection and verification of *mec*C was challenging due to the limited sequence similarity to *mec*A and its common association with a moderate or even low minimal inhibitory concentration (MIC) for oxacillin^5,9–11^. Since then, *mec*C-MRSA were not only found in samples from humans and dairy cattle^5,12^, but also in domestic-, zoo-, and wild animals as well as in wastewater^8,10,13–15^. Human cases of infection associated with *mec*C-MRSA were reported from the United Kingdom, Germany, Austria, Spain and many other European countries^7–9,16^ suggesting that *mec*C-harbouring isolates have become ubiquitous^10,14^.

Mechanisms enabling opportunistic bacteria to cross species barriers, to infect a new host or to enhance their viability in the environment are still poorly understood. Changes expected to be involved in (niche) adaptation processes of *S. aureus* encompass (among others) alterations of metabolic pathways^17^, escape of host defence mechanisms^1^, biofilm formation^18^, iron acquisition abilities^19^, generation of pseudogenes^20,21^ or nucleotide changes altering promoter structures^22^. In addition, production of cytotoxins^23,24^ and other virulence factors as well as their regulation circuits might have a strong influence on host adaptation^23,25,26^. Acquisition of specific virulence factors from a host-specific gene pool^1,27^ by horizontal gene transfer as well as core genome diversification contributes to broadening the host range of *S. aureus*^27^, leading to the definition of extended host spectrum genotypes^28^.

In *S. aureus*, the accessory gene regulator (*agr*) locus represents a quorum sensing system that orchestrates the switch from expressing of surface associated factors (needed for initial attachment) to the expression of exotoxins at high cell density via the effector molecule RNAIII^29^. Notably, *agr* activity appears to be essential for skin and soft tissue infections^30^. Previous research demonstrated the important role of *agr* for virulence factor transcription in animal models of acute infections^31–33^, while *agr* defective mutants seemed to be frequently associated with chronic diseases^34–39^, persistent bacteremia^40^ and cystic fibrosis^41^, indicating adaptation of *S. aureus* to the infected host^42^.

The staphylococcal *agr* locus is an autoinducing control unit that combines bacterial quorum sensing with a classical bacterial two-component regulatory system (TCRS) and the employment of a regulatory RNA (i.e*.* RNAIII) as effector molecule^29^. The locus consists of two divergently arranged transcription units, which are transcribed from promoters P2 and P3, respectively. The P2-derived RNAII harbours the *agrBDCA* operon, while P3 drives transcription of RNAIII. In *S. aureus*, the cell–cell communication (i.e. quorum sensing) depends mainly on autoinducing, short peptides (AIPs) encoded by *agr*D, which are exported by the transmembrane endopeptidase (AgrB) and finally trimmed by a type I extracellular signal peptidase (SspB; reviewed in^43^). At sufficient extracellular AIP concentrations, the peptides binds to the sensor histidine kinases (AgrC) which then phosphorylates the response regulator AgrA (AgrA-P)^43,44^. While a weak baseline transcription from the P2 and P3 promoters is detectable even in the absence of AgrA-P^43,44^, the activated transcription factor strongly (auto)induces expression of both *agrBDCA* and RNAIII^44^. In addition, AgrA-P directly leads to transcription of phenol-soluble modulin (PSM) genes. The majority of *agr*-regulated genes, however, is controlled via RNAIII which influences their transcription and translation^29,43,45^. RNAIII represents a dual-function regulatory RNA that harbours, in addition to its base-pairing functions, a small open reading frame (*hld*) encoding staphylococcal delta-haemolysin (Hld). The *hld* Shine-Dalgarno site was shown to be accessible to ribosomes, indicating that *hld* represents a translatable unit on the RNAIII molecule^46^, which in turn makes Hld detection a suitable proxy to determine RNAIII expression and Agr activity in general.

*S. aureus* virulence often depends on the secretion of large amounts of toxins, including exotoxins with superantigenic functions, i.e., the staphylococcal enterotoxins (SEA, SEB, etc.) and toxic shock syndrome toxin (TSST-1)^47^. Expression of ordinary virulence determinants such as proteases, lipases or nucleases, are promoted by an activated *agr* system, whereby expression of surface binding proteins is downregulated. Nonetheless, this generalized summary still includes exceptions and lineage-specific differences^48^. In addition, primary transcription regulation of virulence factors not encoded by the core genome, e.g. genes associated with the enterotoxin gene cluster (*egc*) harbouring staphylococcal enterotoxins (SE), appears to be less depending on increased *agr* activation^49^.

Biofilm formation is another important feature in *S. aureus* pathogenesis which is influenced by *agr*. The effect of a cellular increase of the *agr* transcript RNAIII, however, is not entirely clear. While previous studies demonstrated enhanced biofilm formation in *agr* deficient strains^42,50^, recent reports, focusing on non-lab-adapted strains, found lineage-specific differences^51^, or a variable role of the *agr* system^38,52^.

Occurrence of independent genomic changes in divergent clonal lineages is generally considered to reflect the adaptation power of bacteria to changing environments^53^. Aim of the study presented here was to analyse the genome structures of emerging *mec*C-MRSA isolates of animal and human origin with the overarching goal to identify genomic patterns that might be associated with the adaptation of such strains to novel hosts and ecological niches. While differences in exotoxin and virulence factor endowments of the isolates were mainly associated with distinct clonal complexes (CC), we found independent genomic variations of the *agr* locus across different *mec*C-MRSA clonal lineages. These changes were associated with varying *agr* activity patterns and kaleidoscopic phenotypes regarding haemolysis and biofilm architecture, suggesting a role of the *agr* system in niche adaptation of the isolates.

## Materials and methods

### Sample collection and pre-screening

*Staphylococcus aureus* isolates obtained from samples of various animal species were collected from January 2014 to December 2016 by IDEXX Laboratories in Ludwigsburg, Germany. Inclusion criteria for isolates were (1) a methicillin resistant phenotype based on growth in the presence of 6 μg/ml cefoxitin according to the manufacturer’s VITEK 2 Advanced Expert System (Nürtingen, Germany) instruction together with (2) oxacillin MICs < 4 µg/ml, as reported for *mec*C-MRSA before^14^. Isolates from seven different European countries were included. Species identity and methicillin resistance mediated by *mec*C was confirmed by PCR as described elsewhere^54^. Antimicrobial susceptibility testing (AST) was carried out using the VITEK 2 system (BioMérieux, Germany) according to the standards given by CLSI VET01-A4 and M100-S24^55,56^. For comparative analysis, additional 12 *mec*C-positive isolates of human origin belonging to corresponding clonal backgrounds collected by the national reference laboratory for staphylococci at the Robert Koch Institute in Germany were included (Table 1).

**Table 1:** Isolate characteristics of 45 *mec*C-MRSA.

ID	Original-ID	Sample source	Host	Country	MLST	*spa*	Oxacillin MIC µg/ml
IMT31818	VB971510	Nose	Dog	Germany	ST599	t278	1
IMT31819	VB971922.1	Uterus	Horse	Germany	ST130	t843	0.5
IMT32509	VB985303	Wound	Cat	Germany	ST599	t278	1
IMT32510	VB992333	Ear	Cat	Poland	ST130	t843	1
IMT32513	VB998882.1	Ear	Cat	Germany	ST599	t16473	1
IMT32929	VB962079.2	Wound	Cat	Netherlands	ST130	t1519	0.5
IMT34478	VB994753.1	Nose	Cat	Germany	ST130	t843	2
IMT34479	VB962798.2	AC	Dog	Germany	ST130	t9165	0.5
IMT34480	VB962790	Ear	Rabbit	Germany	ST130	t9165	1
IMT34485	VB982561.3	Wound	Dog	Germany	ST2361	t10855	1
IMT34488	VB989315	Skin	Cat	Germany	ST130	t843	2
IMT34489	VB993969	Wound	Cat	Germany	ST130	t843	0.5
IMT34491	VB963607.2	Wound	Cat	Germany	ST130	t843	2
IMT36943	VB973587.1	Ear	Cat	Germany	ST599	t278	2
IMT36945	VB972803.2	Frontal sinus	Cat	Italy	ST49	n.a	0.5
IMT36946	VB981317	Wound	Cat	Germany	ST130	t843	2
IMT36947	VB986304	Wound	Cat	Switzerland	ST599	t5930	1
IMT36948	VB987750	TC	Cat	Germany	ST599	t5930	1
IMT36950	VB960116	Skin	Cat	Germany	ST130	t843	2
IMT36952	VB978601	Wound	Cat	Germany	ST2361	t3391	0.5
IMT38113	VB902052	Nose	Cat	Germany	ST2361	t3391	2
IMT38115	VB968721.3	Abscess	Cat	Sweden	ST130	t373	2
IMT38116	VB949481.1	Nose	Hedgehog	Germany	ST599	t278	2
IMT38119	VB972539	Skin	Cat	Germany	ST1764	t524	0.5
IMT39816	PF169945.2	Tissue	Cat	France	ST1245	n.a	0.5
IMT39819	VB945444	Faeces	Sheep	Germany	ST130	t843	2
IMT39820	VB911819.2	Wound	Hedgehog	Germany	ST2361	t3391	2
IMT39824	VB950088.2	Nose	Cat	Germany	ST599	n.a	0.5
IMT39825	VB957945	Nose	Dog	Germany	ST599	t12332	2
IMT40504	BF136317	Nose	Cat	France	ST1943	t8835	1
IMT40506	VB966538	Wound	Cat	Germany	ST130	t843	2
IMT40507	VB966063.1	Wound	Cat	Germany	ST2361	t2345	2
IMT41554	VB999839.3	Wound	Cat	Germany	ST130	t1736	2
RKI5962	14-03729	Ulcus cruris	Human	Germany	ST599	t12332	0.5
RKI5963	13-00970	Wound	Human	Germany	ST599	t5930	0.5
RKI5964	16-02552	Wound	Human	Germany	ST599	t9925	≤ 0.25
RKI5965	14-02098	Abscess	Human	Germany	ST1245	t13902	0.5
RKI5966	15-00967	Wound	Human	Germany	ST130	t14848	0.5
RKI5967	16-01171	Wound	Human	Germany	ST130	t15938	0.5
RKI5968	10-00991	Wound	Human	Germany	ST130	t1736	0.5
RKI5969	13-03754	Wound	Human	Germany	ST2361	t3391	1
RKI5970	18-00258	Infection	Human	Germany	ST2361	t3391	1
RKI5971	18-00326-1	UTI	Human	Germany	ST2361	t3391	1
RKI5972	19-00523	Unknown	Human	Germany	ST49	t208	1
RKI5973	19-00418	Abscess	Human	Germany	ST2361	t2345	0.5

Isolate characteristics of 45 *mec*C-MRSA showing low or moderate oxacillin MICs while being able to grow in the presence of 6 μg/ml cefoxitin.

ID, isolate identification number (this study); Original-ID, original isolate identification number; MLST, multilocus sequence type; *spa*, spa type based on allelic variants of the gene encoding protein A; AC, abdominal cavity; TC, thoracic cavity; UTI, urinary tract infection; n.a., not assigned.

### Whole genome sequencing and genomic analysis of* mec*C-MRSA

MRSA isolates positive for *mec*C were whole-genome sequenced (WGS) using Illumina MiSeq 300 bp paired-end sequencing with an obtained coverage > 90X. Raw reads were used for de novo assembly into contiguous sequences (contigs) and subsequently into scaffolds using SPAdes v3.12^57^. Assembled draft genomes of the isolates were annotated using Prokka^58^. WGS data were used for genotypic characterization including the determination of the sequence type (ST) (MLST v2.0)^59^. Genomic sites of interest and genes encoding for major regulators including the accessory gene regulator (*agr*) system were investigated using Geneious 11.1.5 (Biomatters Ltd., Australia)^60^. Genomic data were further analysed with ResFinder-2.2 (threshold: 90% ID, 80% minimum length), VirulenceFinder-1.6 (threshold: 95% ID, 80% minimum length) and *spa*Typer^59,61–63^.

In order to compare the genomes at high resolution, we used the maximum common genome (MCG) that is defined by those orthologous genes present in all genomes^64^. The coding sequences were clustered based on the parameters of nucleotide sequence similarity (≥ 90%) and gene coverage (≥ 90%). The MCG was defined as those genes that were present in each genome and fulfilled the threshold parameters, yielding 2,094 genes. Allelic variants of these genes were subsequently extracted from all genomes by a blast-based approach, then aligned individually for each gene and concatenated, resulting in an alignment of 1.95 Mbp for these isolates. The alignment was used to generate a maximum likelihood phylogenetic tree using RAxML 8.2.9^65^ which was visualized together with the distribution of the accessory gene content using phandango^66^.

### Phenotypical assessment of accessory gene regulator (*agr*) activity

In this study, complementary phenotypical methods including colony spreading and haemolysin production were employed to investigate the activity of the *agr* system. Synergistic production of different haemolysins (SPDH) on columbia agar plates (Oxoid, Germany) supplemented with 5% sheep blood (SBA) was investigated by cross-streaking tested bacteria perpendicularly to laboratory strain *S. aureus* RN4220 as described before^1^. RN4220 is characterized by a strong β-haemolysin production while α-haemolysin secretion is missing and PSM α peptides are produced only at non-considerable amounts^35, 67,68^. Of note, RN4220 was reported to produce the α-haemolysin encoding mRNA (*hla*), indicating that *hla* transcription cannot necessarily be correlated with α-haemolysin production and/or secretion in *S. aureus*^69^. In addition, the capacity to cause synergistic haemolysis with β-haemolysin is to a large extend due to AgrA-induced expression of PSMs^67^, while β-haemolysin inhibits α-haemolysins’ effect on SBA^35^. After overnight incubation of the plates at 37 °C, the haemolysis zones were examined and pictures were taken.

A modified CAMP test for verification of β-haemolysin production was performed using *Streptococcus agalactiae* (ATCC12386) and the β-haemolysin producing *S. aureus* strain ATCC25923 (positive control) as described recently^1^. Briefly, staphylococci of interest were streaked perpendicularly to CAMP-factor producing ATCC12386. A positive interaction of CAMP-factor from Group B streptococci with β-haemolysin (phospholipase C) of *S. aureus* is characterized by completely lysed sheep blood erythrocytes forming a clear semilunar-shaped area^70^.

We also performed a colony spreading assay on soft agar described by Kaito et al.^71^, since an activated state of the *agr* system^72^ as well as AgrA-depending production PSM α3 are necessary for *S. aureus* to slide on wet surfaces^73^. Beyond that, a mutation in the membrane-bound transpeptidase sortase A (SrtA), or for instance the lack of its substrates fibronectin binding protein A and B (FnBPA, FnBPB), clumping factor A and B (ClfA, ClfB) enhances spreading^74^. Briefly, 20 ml tryptic soy soft agar plates (0.24%) were used to investigate spreading activity of all MRSA. After overnight incubation of the plates at 37 °C, the spreading zones were examined, and pictures were taken.

The standard laboratory strains USA300 (FPR3757), the USA300 *agr*A::Tn mutant (NE1532) and RN4220 were included in all phenotype assays as controls for wild type (wt) *agr* functionality, a loss-of-function- and a functionally impaired *agr* system, respectively^69^. In lab strain RN4220, a frameshift mutation in the *agr*A-coding region adds three amino acids to the C-terminus of AgrA, a variation known to cause a considerable delay in RNAIII transcription compared to the wild type^70^. To verify production of TSST, the TST-RPLA Kit TD940 (Thermo Fisher Scientific, USA) was used according to the manufacturer’s instructions.

All surveys were repeated thrice for each of the isolates including the reference strains.

### Proteomic analysis of α-, β- and δ-haemolysin production in* mec*C-MRSA

Protein abundance values for Hla/Hlb/Hld from whole bacterial cell preparations were measured to assess RNAIII transcription capabilities by Hld production and Hla and Hlb (pre-) proteins of the bacteria and their general ability to produce them.

### Sample preparation by easy extraction and digestion (SPEED)

Samples were prepared in triplicates using SPEED as previously described^75^. In brief, cells were re-suspended in trifluoroacetic acid (TFA) (Uvasol for spectroscopy, Merck, Darmstadt, Germany) (sample/TFA 1:4 (v/v)) and incubated at 70 °C for 3 min. Samples were neutralized with 2 M TrisBase using 10 × volume of TFA and further incubated at 95 °C for 5 min after adding Tris(2-carboxyethyl)phosphine (TCEP) to a final concentration of 10 mM and 2-Chloroacetamide (CAA) to a final concentration of 40 mM. Protein concentrations were determined by turbidity measurements at 360 nm, adjusted to 0.25 µg/µl using a 10:1 (v/v) mixture of 2 M TrisBase and TFA and then diluted 1:5 with water. Digestion was carried out for 20 h at 37 °C using Trypsin (Promega, Fitchburg, WI, USA) at a protein/enzyme ratio of 50:1. Resulting peptides were desalted using StageTips C18^76^.

### Liquid chromatography and mass spectrometry

Peptides were analyzed on an EASY-nanoLC 1,200 (Thermo Fisher Scientific, Bremen, Germany) coupled online to a Q Exactive™ Plus mass spectrometer (Thermo Fisher Scientific, Bremen, Germany). 1 µg peptides were loaded on an Acclaim PepMap trap column (20 mm × 75 μm i.d., 100 Å, C18, 3 μm; Thermo Fisher Scientific, Bremen, Germany) and were subsequently separated on a 200 cm μPAC column (PharmaFluidics, Ghent, Belgium). The flow rate was set to 800 nl/min and a stepped 40 min gradient was applied: 3–10% B in 4 min, 10–33% B in 17 min, 33–49% B in 4 min, 49–80% B in 7.5 min and 80% B for 7.5 min. Solvent A was 0.1% (v/v) formic acid (FA) in water, solvent B consisted of 80% (v/v) acetonitrile in 0.1% (v/v) FA.

The Q Exactive Plus was operated in data-independent (DIA) manner in the m/z range of 350–1,150. Full scan spectra were recorded with a resolution of 70,000 using an automatic gain control (AGC) target value of 3 × 10^6^ with a maximum injection time of 100 ms. The Full scans were followed by 53 DIA scans of dynamic window widths using an overlap of 0.5 Th (Supplemental Table 1). DIA spectra were recorded at a resolution of 17,500@200 m/z using an AGC target value of 3 × 10^6^ with a maximum injection time of 55 ms and a first fixed mass of 200 Th normalized collision energy (NCE) was set to 25% and default charge state was set to 3.

### Mass spectrometric data analysis

The mass spectra were searched in DIA-NN (Version 1.7.6)^77^ using the deep-learning based spectra and RT prediction for sequences from the complete proteome of *S. aureus* strain NCTC 8,325 (UP000008816, 2,889 sequences, downloaded 4/10/18) and sequences of selected genes (*hla*, *hlb* and *hld*) obtained from whole genome sequencing. Spectra were searched with a tolerance of 10 ppm in MS^1^ and 20 ppm in MS^2^ mode, strict trypsin specificity (KR not P) and allowing up to one missed cleavage site. Cysteine carbamidomethylation and N-terminal methionine excision were set as modifications. Peptide length was restricted to 7–30 amino acids. The m/z ranges were 350–1,150 for full scans and 200–1,800 for DIA scans. A false discovery rate of 1% was applied for precursor and protein identifications.

### Biofilm formation assay on polystyrene tissue culture plates

Biofilm formation to inert artificial surfaces was tested in 96‐well polystyrene tissue culture plates (Greiner Bio‐One, Cellstar, F‐form) as described previously^78^. Briefly, *S. epidermidis* RP62A and *S. carnosus* TM300 were used as positive and negative controls, respectively. Bacterial overnight cultures in triplicates were grown in Trypticase Soy Broth (TSB; Becton Dickinson) which contains, according to the standard composition, 2.5 g/l glucose. Cultures were diluted in fresh TSB to an OD_600_ of 0.05 and 200 µl filled in each well (four wells per biological replicate) and incubated under static condition at 30 °C for 18 h. Supernatant was discarded and adherent cells were washed twice with 1 × PBS buffer before the remaining cells were heat-fixed at 65 °C for 1 h. Plates were then stained with 10 mg/ml crystal violet for 2 min, washed twice with double-distilled water before proceeding with measuring the absorbance at OD_492_ by an ELISA plate reader (Multiskan Ascent).

### Imaging of the biofilm architecture by confocal laser scanning microscopy (CLSM)

In order to study the putative effect of different *agr* non-wt variants on growth/biofilm characteristics on glass surfaces, overnight cultures of closely related isolates were subjected to comparative analysis: *S. aureus* isolates belonging to CC130 IMT38119 (*agr* III wt), RKI5966 (*agr *III non-wt AgrC variant) and IMT31819 (*agr *III non-wt AgrA variant) were diluted to 10^5^ bacteria per ml. One milliliter diluted suspension was used to inoculate the wells of a 24-well plate with glass bottom (µ-Plate 24 Well Black, ibidi GmbH, Germany), which was then cultivated for 20 h at 37 °C with 150 rpm on an orbital shaker. Afterwards, samples were photographed (Lumix GM1, Panasonic, Japan) on a light table, stained with LIVE/DEAD (LIVE/DEAD Cell Viability Assay, ThermoFisher Scientific, Germany) according to the manufacturer’s instructions and imaged with a confocal laser scanning microscope (LSM780, Carl Zeiss AG, Germany) using the Plan-Apochromat 20 × /0.8 objective.

When necessary, images were cropped, adjusted for optimal brightness and contrast (applied to the whole image) using Adobe Photoshop CS6 (Adobe Systems, San Jose, CA, USA).

### Database accession numbers

Genomic sequencing data used are available for download from the National Center for Biotechnology Information (NCBI) under BioProject accessions PRJNA588740. Accession numbers of whole genomes sequences are provided in Supplemental Table 2. The mass spectrometry proteomics data have been deposited to the ProteomeXchange Consortium (https://proteomecentral.proteomexchange.org) via the PRIDE partner repository with the dataset identifier PXD016486.

## Results

### General features of the *mec*C-MRSA strain collection

**Figure 1:**
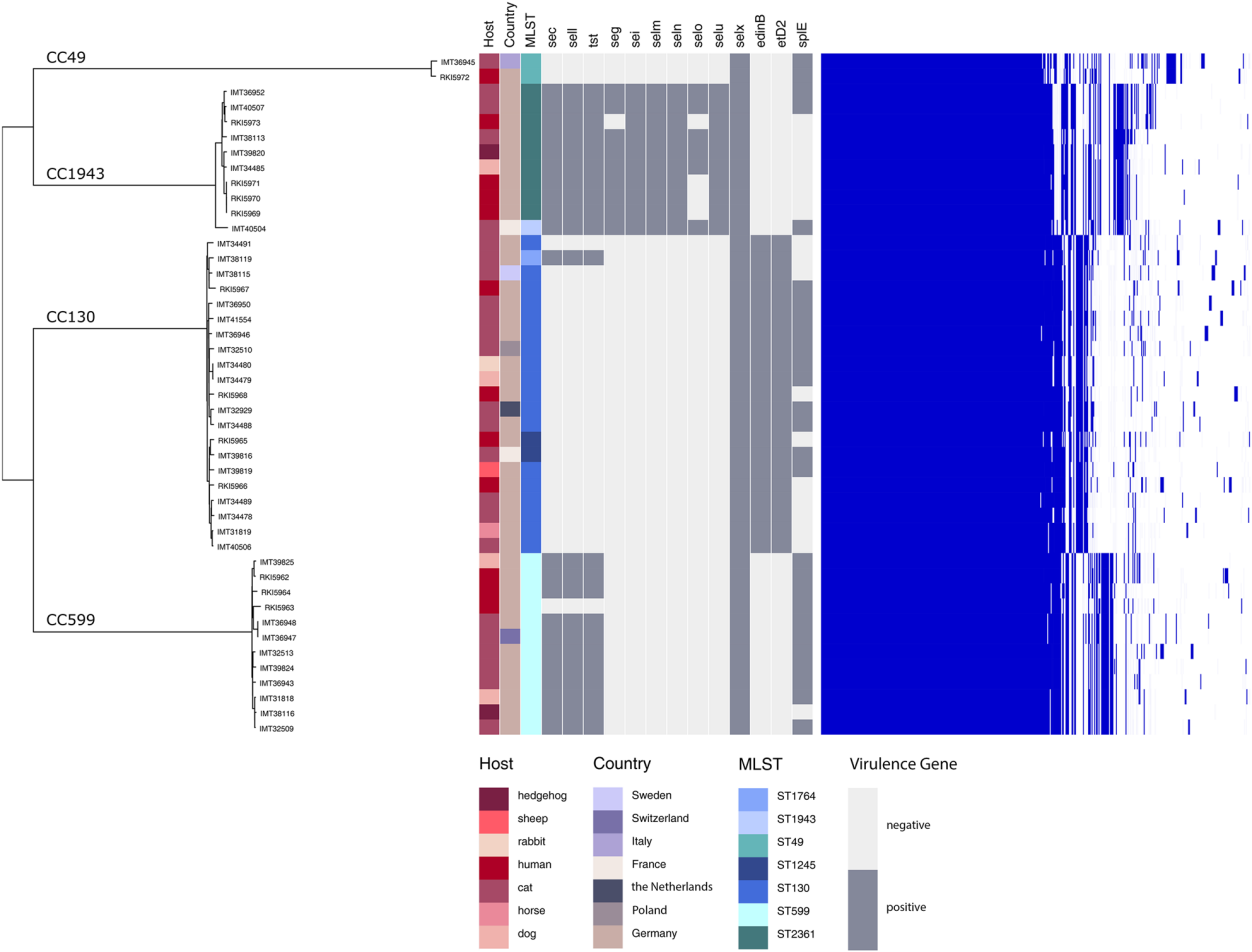
The core genome phylogeny based on the Maximum Common Genome. The core genome phylogeny based on the Maximum Common Genome comprising 2,094 orthologous genes present in all isolates show four distinct clusters, whereby the genetic diversity within the clusters is rather low. Furthermore, the isolates metadata show no significant association with the core genome clusters. The 2,003 accessory genes show a distribution pattern that is highly correlated with the core genome clusters (right side), suggesting a lineage-specific gene content. Genes for aureolysin (*aur*), leucotoxins D and E (*luk*D, *luk*E), gamma-haemolysin component A–C (*hlg*A, *hlg*B, *hlg*C) and proteases SpIA or SpIB are present in all isolates. All isolates belonging to ST-1943 as well as some CC130 and CC599 were positive for different variants of the Staphylococcal pathogenicity island (SaPI) harbouring a toxic shock toxin encoding gene (*tst*), which were variants of *tst-*bov^80^. Moreover, 48.5% of the *mec*C-positive isolates harboured staphylococcal enterotoxins (SE). The protease SpIE can just be found in 23/33 isolates and is not associated with any sequence type. The epidermal cell differentiation inhibitor B (*edin*B) cannot be determined in the isolates of ST-1943, ST-2361, ST-49 and ST-599.

A total of 33 *mec*C-MRSA were obtained from seven different European countries and six different animal species for whole genome sequencing (WGS). Isolates from cats dominated the collection (24/33), followed by those obtained from dogs (4/33) and other companion-, wild- and livestock animals (Table 1). We also included 12 isolates from human patients belonging to matching genotype lineages (Table 1, Fig. 1) for further phenotype assays and subsequent comparative genome analysis. All 45 isolates displayed low or moderate oxacillin MICs < 4 µg/ml, while being able to grow in the presence of 6 μg/ml cefoxitin (Table 1), which is typical for *mec*C-MRSA^14, 79^. AST results revealed additional resistance to aminoglycosides (gentamicin, kanamycin) for isolate RKI5962 only. A positive latex agglutination test verified production of the TSST toxin for all isolates harbouring a *tst-*bov variant^80^. Interestingly, we noticed considerable phenotypic differences regarding haemolysis on SBA between the isolates, even among those sharing the same phylogenetic background. This prompted further investigations into putative genomic changes which might account for these variations.

### Phylogenetic relationship and distribution of virulence-associated factors among *mec*C-MSRA

WGS of the isolates revealed that the 45 *mec*C-positive MRSA belonged to four CCs: CC599 (12), CC130 (21), CC1943 (10) and CC49 (2) (Table 1, Fig. 1). Pairwise SNP-distances between the core genomes were calculated for all MRSA isolates (Supplemental Table 3), showing a very close phylogenetic relationship of those genomes belonging to the same CC. While the differences within one clonal complex ranged between 100 and 500 SNPs, the genomes belonging to different CCs differed by more than 10,000 SNPs, resulting in a clear clustering of MRSA from the same CC (Fig. 1). Metadata such as geographic origin, disease or animal species showed no significant association with the core genome clustering, but a lineage-specific association of the variable gene content was obvious (Fig. 1).

Nevertheless, a core set^81^ of *S. aureus* virulence factors was present in all isolates, including aureolysin (*aur*), bi-component leukotoxin (*luk*D/E), γ-haemolysin components A–C (*hlg*A, *hlg*B, *hlg*C) and genes encoding for the serine proteases SpIA and SpIB. Presence of the epidermal cell differentiation inhibitor B (*edin*B) and exfoliative-like toxin D2^82^ was associated with CC130. When considering factors promoting biofilm production in *S. aureus*, all isolates harbour complete *ica* loci (*ica*R/*ica*ADBC) but not the biofilm-associated surface protein *bap*. For iron acquisition, all isolates harboured amongst others the iron-regulated surface determinant gene cluster *isd*ABCDEF.

While only one isolate lacked the gene encoding α-haemolysin (*hla*) completely, three isolates belonging to CC130 carried *hla* variations which resulted in aa sequence alterations and two further isolates harboured insertions (details are provided in Supplemental Table 4). Bacteriophages converting the β-haemolysin gene (*hlb*) were identified in four isolates (for details see Supplemental Table 4). Some of the *mec*C-MRSA, especially those belonging to CC599 and CC1943 harboured variants of previously described staphylococcal pathogenicity islands, including SaPI*bov*^80^ encoding SEC, a TSST*bov* variant and SElL (Fig. 1). Isolates belonging to CC1943 were positive for an *egc* cluster variant (*seg*, *sei*, *selm*, *seln*, *selo*, *selu*), while all isolates were positive for *sel*X (Fig. 1). Furthermore, all isolates were positive for intact and identical genes encoding phenol-soluble modulins (PSM α1 to PSM α4 and PSM β1 and PSM β2), and differences in the promoter regions of these operons seemed to particularly mirror the genomic lineage (data not shown).

### *mec*C-MRSA belonging to CC599, CC49, CC130 and CC 1943 harbour* agr *variations

**Figure 2:**
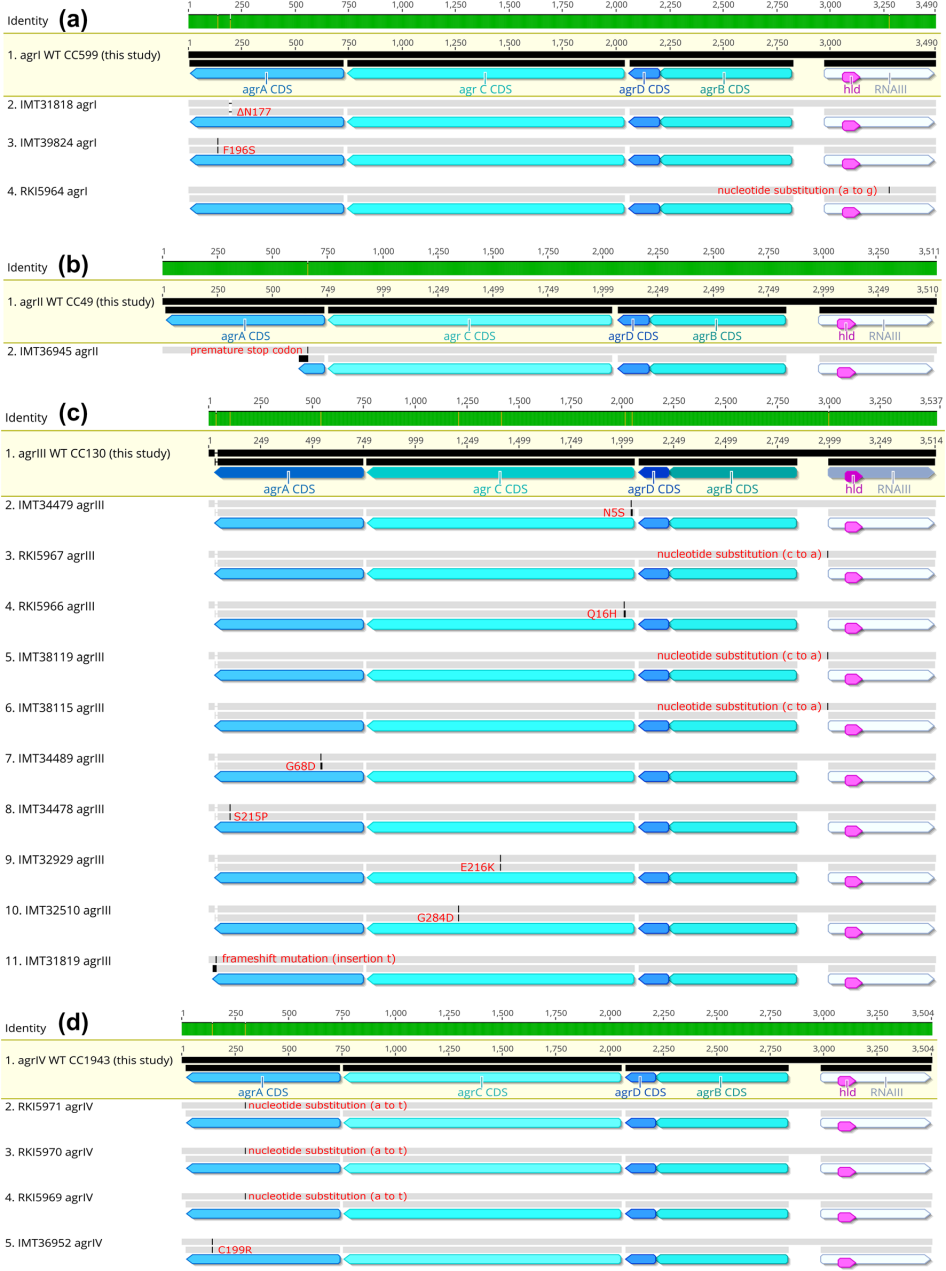
Sequence alignments of *agr* regions in *mec*C-MRSA. Sequence alignments for (**a**) CC599 (*agr* I), (**b**) CC49 (*agr* II), (**c**) CC130 (*agr* III) and (**d**) CC1943 (*agr* IV) isolates. First row shaded in yellow indicates the wild type (wt) exemplarily shown for all isolates sharing 100% coverage and 100% nucleotide and amino acid sequence identity. For each of the non-wt isolates on the display, changes within the upper gray line indicates a nucleotide sequence alteration while changes in the second line indicates amino acid sequence alteration. For all details and the reference sequences used for each CC see Supplemental Table 4. (**a**) First row, wt *agr* I in CC599 shared by IMT32509, IMT32513, IMT36943, IMT 36,947, IMT36948, IMT38116, IMT39825, RKI5962, RKI5963 and RKI5964. Second row, IMT31818 has a triplet nucleotid deletion resulting in ΔN177 in AgrA; third row, IMT39824 shows a non synonymous substitution (from A to G in position 289) leading to F196S in AgrA. (**b**) First row, wt *agr* II in CC49 represented by RKI5972 while the second row shows IMT36945 with an insertion creating a premature stop codon in AgrA. (**c**) First row, wt *agr* III in CC130 shared by IMT34480, IMT34488, IMT34491, IMT36946, IMT36950, IMT38115, IMT38119, IMT39816, IMT39819, IMT40506, IMT41554, RKI5965, RKI5967 and RKI5968. Rows 2, 4, 9 and 10 show amino acid variations in AgrC for IMT34479 (N5S), RKI5966 (Q16H), IMT32929 (E216K) and IMT32510 (G284D) generated by corresponding non-synonymous substitutions. RKI5967 (row 3), IMT38119 (row 5) and IMT38115 (row 6), harbour a nucleotide substitution (**c** to **a**) at position -2 upstream the RNAIII sequence start, respectively. Rows 7, 8 and 11 harbour variants of AgrA for IMT34489 (G68D), IMT34478 (S215P) caused by non-synonymous substitutions and IMT31819 shows an insertion at position 711 bp in *agr*A causing an alternate stop codon. (**d**) First row, WT *agr* IV in CC1943 shared by RKI5973, IMT34485, IMT40507, IMT40504, IMT38113. Second to fourth row (upper gray line) show a synonymous substitution (a to t at position 442) in *agr*A for RKI5971, RKI5970 and RKI5969 while the fifth row indicates a further variation of *agr*A (C199R) generated by a non-synonymous substitution.

According to the typing scheme used by Shopsin et al.^83^, CC599 MRSA were assigned to the *agr* type I, CC49 to type II, CC130 to type III and CC1943 to type IV. Of the isolates harbouring the type I *agr* system, two isolates showed aa changes for AgrA and all AgrB aa sequences showed the variation A182T when compared to the *S. aureus* strain N315 sequences (Supplemental Table 4), while the respective aa sequences for AgrC and AgrD were identical (Fig. 2). All isolates belonging to CC599 and CC49 showed a nucleotide insertion (T) at bp 55 within the region encoding RNAIII, resulting in 514 + 1 bp length and an additional uracil (U) in the hairpin 2, according to the secondary structure model of RNAIII proposed by Benito and colleagues^46^. Since this insertion was found in all isolates associated with the *agr* types I and II in this study, this variation was considered as the wild type (wt) sequence here. Only two isolates belonged to the *agr* type II, and variation among them was detected in terms of a premature stop codon created by an insertion in the AgrA encoding gene in isolate IMT36945 (Fig. 2). Most variants were detected among the aa sequences for AgrA (one insertion, two changes) and AgrC (four changes) of the CC130 isolates harbouring the type III *agr* system (Fig. 2, and Supplemental Table 4). For the CC1943 isolates (type IV *agr*), only IMT36952 showed an aa sequence variation in AgrA (Fig. 2). The nucleotide sequence regions encoding for the 26-aa δ-haemolysin integrated in RNAIII and the promoter sequences for RNAII (P2) and RNAIII (P3) were conserved in all 45 genomes investigated here (Fig. 2a–d).

### Assessment of genes associated with virulence factor transcription in* mec*C-MRSA

Only in isolate IMT41554 (CC130), a C to T change within the − 35 promoter region previously identified as the AgrA binding region of the *psm* α operon^84^ was detected at position − 27. As the phenotype did not deviate from closely related isolates, this single nucleotide change is unlikely to affect the AgrA-binding abilities upstream of *psm* α1–4 in IMT41554. Yet, many two component regulatory systems (TCRS) and nucleic acid-binding proteins seem to modulate *S. aureus* virulence factor expression, especially of exotoxins with haemolytic activity^85^. Since TCRS variations might influence the particular phenotype appearance of *S. aureus*, the isolate collection was screened for obvious deletions, insertions or changes generating for instance amino acid (aa) substitutions or premature stop codons. Among the TCRS included in the analysis were those either positively or negatively influenced by *agr* such as the staphylococcal accessory regulator nucleic acid-binding protein (SarA) and the regulator of toxins (Rot). Overall, amino acid changes (referred to the respective reference sequence) in global regulators were rare and mostly lineage-specific (Supplemental Table 5), indicating a limited role of these variations for the phenotype differences observed for each CC.

### Haemolysis and colony spreading of *mec*C-MRSA carrying* agr* variations

While analysing the *mec*C-MRSA belonging to four different phylogenetic lineages, we noticed a broad range of different phenotypes with respect to haemolysis on SBA plates.

**Figure 3:**
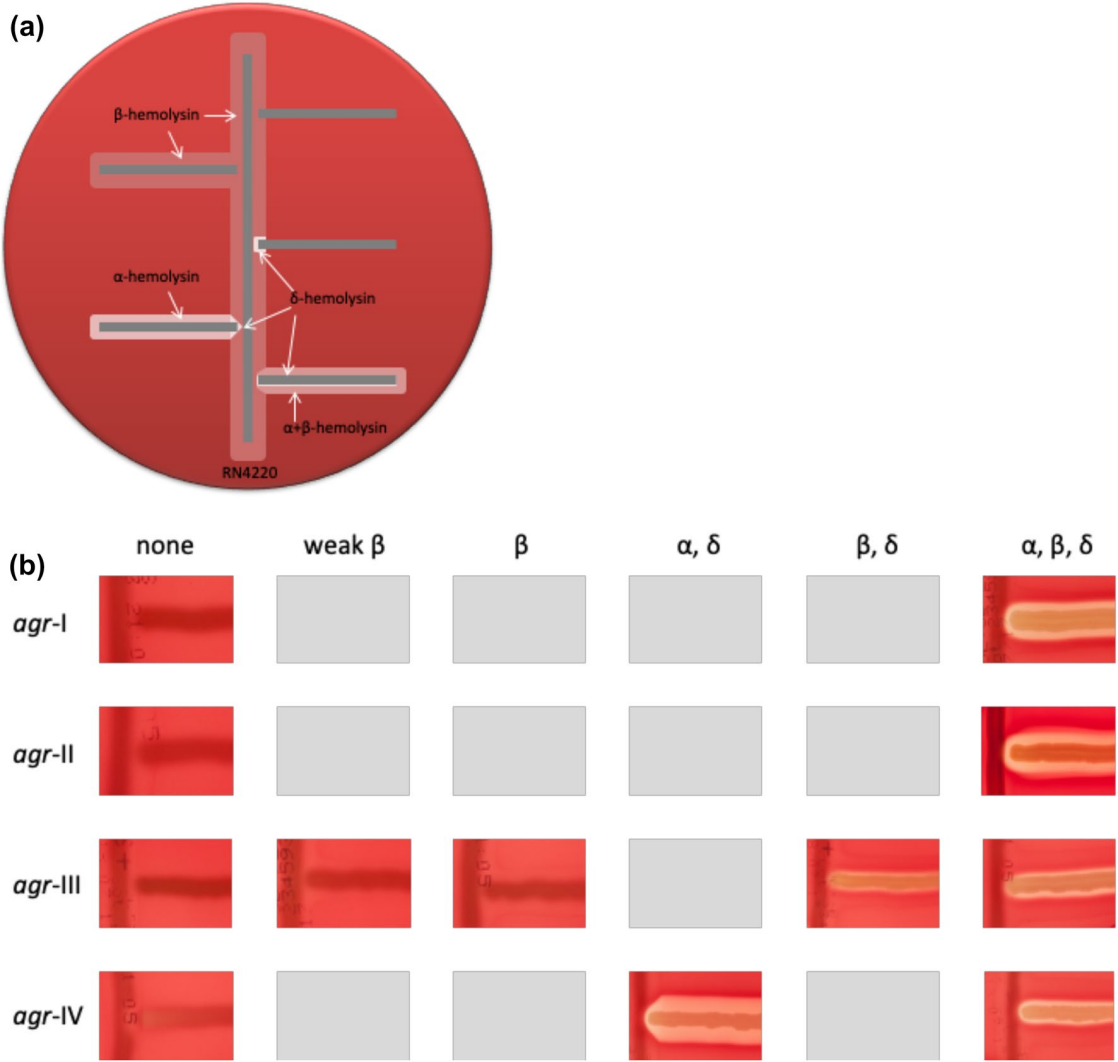
Haemolytic activities of *S. aureus* isolates on sheep blood agar (SBA) plates. (**a**) Scheme for assessment of haemolytic activity based on Geisinger et al.^118^. The isolates were tested by *cross-streaking perpendicularly* to *S. aureus RN4220* on sheep blood agar (SBA) plates. The turbid zone induced by β-haemolysin production of RN4220 enhanced lysis by δ-haemolysin and PSMs (clear zone at the intersection) and inhibited α-haemolysin (V-shaped zone at the intersection). (**b**) Haemolytic activity of *mec*C-positive *S. aureus* belonging to different *agr* types on SBA plates. The lack of a corresponding phenotype in the isolate collection is indicated by a grey rectangle. One exemplarily image was used to illustrate the differences, respectively.

**Figure 4:**
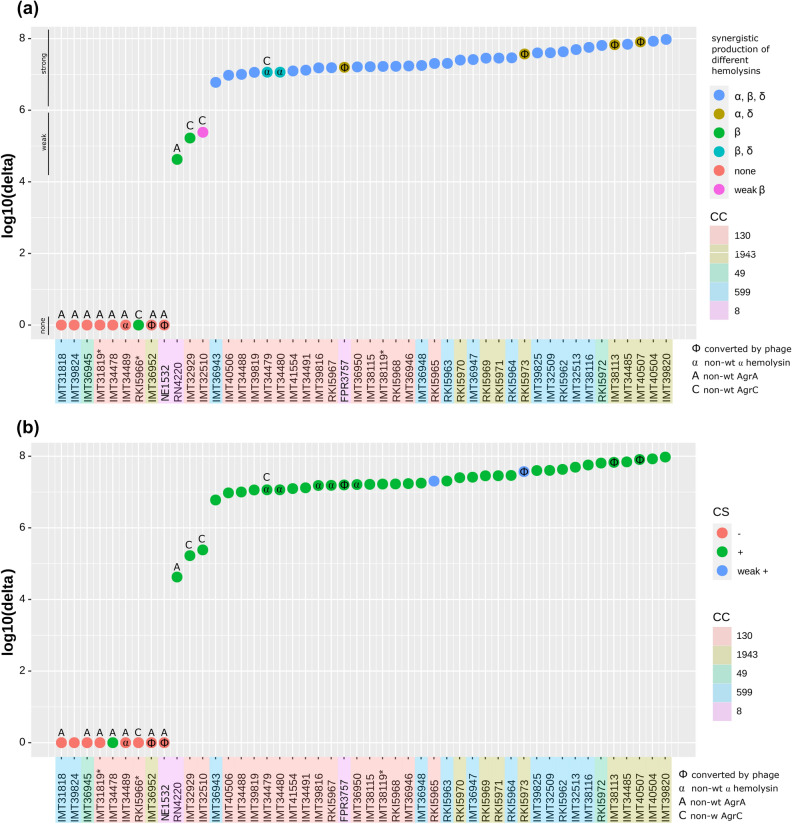
Illustration showing protein abundance values, relevant genomic variation and phenotype results of *mec*C-MRSA. (**a**) δ-haemolysin (Hld) protein abundance and the synergistic production of different haemolysins (SPDH test). (**b**) δ-haemolysin (Hld) protein abundance and the isolates’ capability for colony spreading.

Initially, haemolytic activities were assessed using a SPDH cross-streaking test utilizing strain RN4220 (Fig. 3). Here, only 30 of 45 isolates showed the typical haemolysis pattern for α-, β- and δ-haemolysin production (Fig. 4, Supplemental Figures 1 and 2), suggesting that some of the isolates might harbour genomic alterations affecting haemolytic activities (Table 2). We then used the CAMP test which indicates secretion of β-haemolysin by *S. aureus* through a characteristic arrow-shaped synergistic haemolysis zone on SBA. 10/45 isolates and two of the reference strains failed to produce the corresponding phenomenon (Table 2, Supplemental Figure 1), with four isolates (and two reference strains) harbouring a phage disrupting the *hlb* gene (Supplemental Table 4). The remaining six isolates lacked the *hlb*-converting phage but showed instead AgrA variations that clearly deviated from the wildtype (Fig. 2, Supplemental Table 4).

**Table 2:** Phenotypic characterisics of *mec*C-MRSA.

ID	CC	*agr*	CS	Haemolysis on SBA		BF	*agr* acitivity level (predicted)
				CAMP	SPDH		
**IMT31818**	599	I	−	−	None	0	0
IMT32509	599	I	+	+	α, β, δ	0	++
IMT32513	599	I	+	+	α, β, δ	0	++
IMT36943	599	I	+	+	α, β, δ	0	++
IMT36947	599	I	+	+	α, β, δ	1	++
IMT36948	599	I	+	+	α, β, δ	1	++
IMT38116	599	I	+	+	α, β, δ	0	++
**IMT39824**	599	I	−	−	None	0	0
IMT39825	599	I	+	+	α, β, δ	0	++
RKI5962	599	I	+	+	α, β, δ	0	++
RKI5963	599	I	+	+	α, β, δ	0	++
**RKI5964**	599	I	+	+	α, β, δ	0	++
**IMT36945**	49	II	−	−	None	0	0
RKI5972	49	II	+	+	α, β, δ	0	++
IMT31819*	130	III	−	−	None	0	0
**IMT32510**	130	III	+	Weak +	Weak β	0	++
**IMT32929**	130	III	+	Weak +	β	1	++
**IMT34478**	130	III	+	−	None	1	0
**IMT34479**	130	III	+	+	β, δ	1	++
IMT34480	130	III	+	+	β, δ	1	++
IMT34488	130	III	+	+	α, β, δ	0	++
**IMT34489**	130	III	−	−	None	0	0
IMT34491	130	III	+	+	α, β, δ	0	++
IMT36946	130	III	+	+	α, β, δ	0	++
IMT36950	130	III	+	+	α, β, δ	0	++
**IMT38115**	130	III	+	+	α, β, δ	0	++
IMT38119*	130	III	+	+	α, β, δ	0	++
IMT39816	130	III	+	+	α, β, δ	0	++
IMT39819	130	III	+	+	α, β, δ	2	++
IMT40506	130	III	+	+	α, β, δ	1	++
IMT41554	130	III	+	+	α, β, δ	1	++
RKI5965	130	III	Weak +	+	α, β, δ	1	++
RKI5966*	130	III	−	+	β	0	0
**RKI5967**	130	III	+	+	α, β, δ	0	++
RKI5968	130	III	+	+	α, β, δ	1	++
IMT34485	1943	IV	+	+	α, β, δ	0	++
**IMT36952**	1943	IV	−	−	None	2	0
IMT38113	1943	IV	+	−	α, δ	0	++
IMT39820	1943	IV	+	+	α, β, δ	0	++
IMT40504	1943	IV	+	+	α, β, δ	0	++
IMT40507	1943	IV	+	−	α, δ	0	++
**RKI5969**	1943	IV	+	+	α, β, δ	0	++
**RKI5970**	1943	IV	+	+	α, β, δ	0	++
**RKI5971**	1943	IV	+	+	α, β, δ	0	++
RKI5973	1943	IV	Weak +	−	α, δ	2	++

Genomic variation within the *agr* encoding region is indicated by use of bold ID letters (for details see Fig. 2). The isolates further investigated with respect to biofilm structures using CLSM are marked with *. Agr activity prediction according to Hld values presented in Fig. 4a (0, +, ++).

ID, isolate number; CC, clonal complex; *agr*, accessory gene regulatory type; CS, colony spreading (phenotypical verification for *agr* functionality); CAMP, phenotypical verification for β-haemolysin production; SPDH, test results for synergistic production of different heamolysins using RN4220 (phenotypical verification of α-, β- and δ-haemolysin production); BF, biofilm formation (mean absorbance 492 nm; values: 0, < 0.2811; 1, 0.2811 < x < 1.0; 2, > 1.0); + , positive; −, negative.

**Figure 5:**
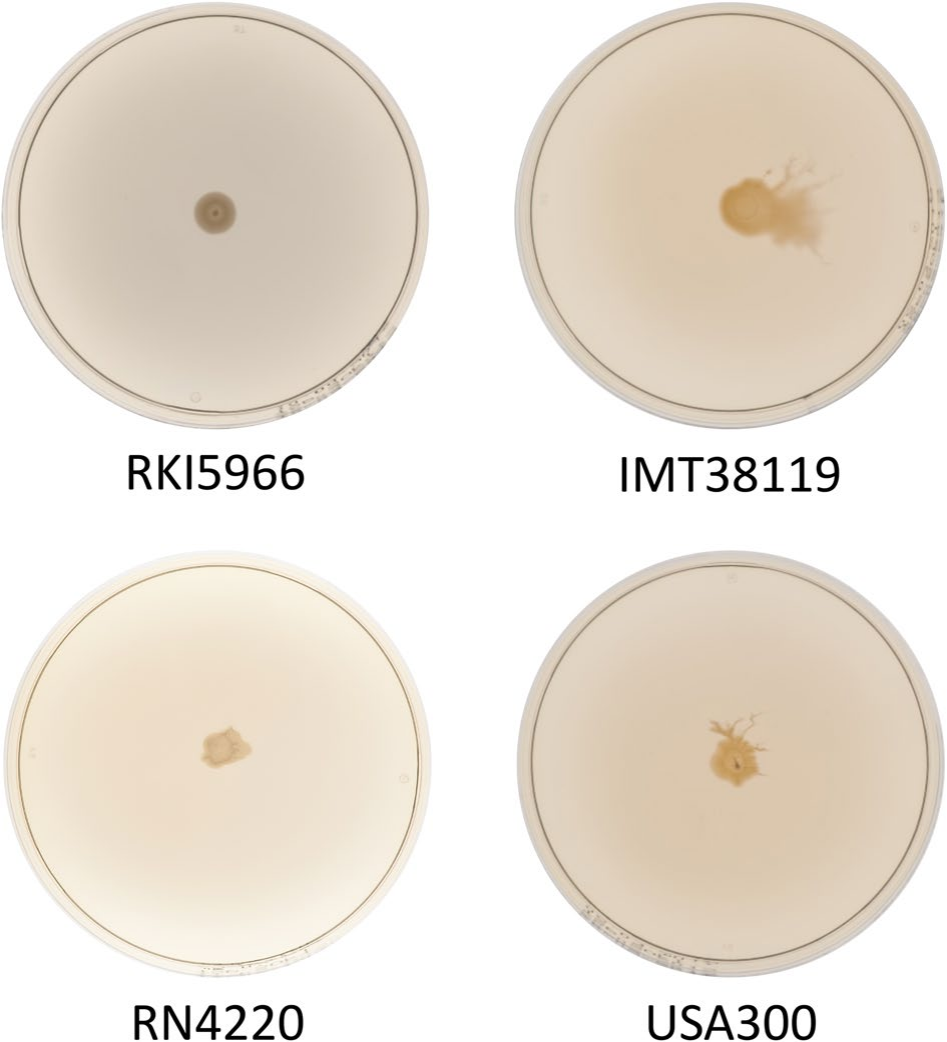
Colony spreading assay results for *mec*C-*S. aureus* with different *agr* functionalities on soft agar plates. A TSA soft agar plate (0.24%) was inoculated with 2 µl overnight culture of *S. aureus*. Examples shown here include isolate RKI5966 associated with a weak *agr* functionality, which was not able to spread on semisolid agar plates, while isolate IMT38119 (strong *agr* functionality) showed spreading. As controls we have employed the standard laboratory strains RN4220 (weak *agr* functionality) and USA300 (strong *agr* functionality).

Since functionality of the *agr* system is also reflected by the ability of *S. aureus* to slide over wet surfaces^72^, we performed a colony spreading assay on semisolid agar plates. As a result, 38/45 isolates showed the characteristics of “sliding bacteria”, which are exemplarily shown in Fig. 5. A comprehensive summary of all phenotype characteristics of the isolate collection and the reference strains included is presented in Table 2.

### *Agr *activity assessment by measuring δ-haemolysin production in *mec*C-MRSA using proteomics

The protein abundance of Hld (δ-haemolysin) was used to assess RNAIII transcription capabilities of *S. aureus* isolates, as the corresponding coding gene (*hld*) is integrated in the RNAIII transcript. Hld abundance was measured using whole cell proteomics to cover the actual expression level. Therefore, the assessment of direct whole-cell δ-haemolysin production appears as a suitable method to predict RNAIII transcription, as long as occurring sequence variations do not affect the *hld* encoding gene or the promoter regions in in the *agr* operon (Figs. 2 and 4a).

The protein abundance levels for δ-haemolysin measured by mass spectrometry are shown in Supplemental Table 6, and putative CC-specific differences were noticed (Fig. 4a). Isolates sharing the CC1943 background and lacking *agr* alterations, for example, showed higher overall δ-haemolysin abundance values than those belonging to CC130 or CC8 (Supplemental Table 6, Fig. 4a). Based on the δ-haemolysin detection level, we categorized the individual isolates’ *agr* activity as follows: 0, lack of detectable *agr* activity; +, weak *agr* activity and ++, strong *agr* activity (Fig. 4a). The reference strains included showed Hld abundances which clearly correlate with their previously reported *agr* functionality: For RN4220, a delay towards production of Hld due to an *agr*A mutation is known^86^. This alteration is also mirrored by a decreased protein abundance (Fig. 4a) and was consequently considered as “weak” *agr* activity. Moreover, the USA300 *agr*A::Tn strain (NE1532) completely lacked Hld detection, as expected. Thus, detection of Hld production together with a genomic inspection of the *agr* encoding region is indeed suitable to predict RNAIII transcription and *agr* functionality.

### Comparison of phenotype assay results and haemolysin abundances

We then plotted the proteomic data against the results obtained from the phenotype assays described above.

The δ-haemolysin protein abundance and haemolysis phenotype associated with the isolates and reference strains are shown in Fig. 4a. Strikingly, all isolates (and reference strains) showing a strong reduction or lack of δ-haemolysin production were found to carry non-wt variants of either AgrA or AgrC (Fig. 4a). In addition, these AgrA or AgrC non-wt variants were associated with non-wt haemolysis patterns, too (Table 2, Supplemental Table 4, Fig. 4a).

The results for β-haemolysin (Hlb) protein abundances and the isolates’ capability to produce a synergistic haemolysis (CAMP phenomenon) are presented in Supplemental Figure 2 and Table 2, with isolates harbouring phage-disrupted *hlb* genes and *agr* alterations being indicated. Interestingly, all isolates with non-wt AgrA variants also failed to produce the CAMP phenomenon, even when whole-cell β-haemolysin abundance levels (e.g. for IMT36945) were obviously sufficient to induce the phenotype in matching *agr* wt isolates (e.g. RKI5972). Two further isolates harbouring non-wt variants of AgrC (IMT32510 and IMT32929) produced only a weak CAMP phenomenon (blue dots in Supplemental Figure 2). Thus, a functionally active *agr* system seems to be necessary for *S. aureus* to produce the exhibit CAMP phenomenon on SBA.

When comparing the results of whole-cell α-haemolysin detection with the haemolysis phenotypes obtained by the SPDH test we noted considerable discrepancies. Thus, we clearly found Hla protein production in some of the isolates harbouring AgrA and AgrC variations, but the strains did not exhibit an α-haemolysin phenotype on SBA (Fig. 3 and Supplemental Figure 1). Vice versa, in five isolates showing α-, β-, δ-haemolysin activity on SBA (blue dots in Supplemental Figure 1), no Hla protein levels were detectable. This could for instance be a result from repeated mis-judgement of the phenotype, from significant differences for Hla abundances between proteome- and secretome or the detection limit of the mass spectrometry. Of note, the α-haemolysis phenotype was absent in all isolates harbouring AgrA or AgrC variants (Supplemental Figure 1), again indicating that a functional *agr* system is required for inducing an α-haemolysis zone on SBA.

Finally, colony spreading on wet surfaces is known to be directly induced by AgrA-P. As shown in Table 2, all isolates lacking this feature displayed non-wt variants of AgrA or AgrC, while some of the isolates with AgrA or AgrC variants were still capable to spread (Figs. 4b,5).

### *Agr *variation is not associated with differences in biofilm formation on polystyrene tissue culture plates

All 45 *mec*C-MRSA isolates were tested for their ability to form stable biofilms on the inert surfaces of polystyrene tissue culture plates in a standard crystal violet-staining assay. The test allows for the detection and quantification of sturdy biofilms that are firmly attached to artificial surfaces and which are not removable by washing. With respect to the 12 isolates belonging to CC599, only two (IMT36947 and IMT36948) formed a visible biofilm (value-1-biofilm) in the crystal violet assay (Table 2, detailed information in Supplemental Table 7). Among the 21 CC130 isolates, 12 were biofilm negative (value-0-biofilm), eight displayed moderate biofilm levels (value-1-biofilm) and one isolate produced a strong biofilm (value-2-biofilm). In addition, two out of ten CC1943 *mec*C-MRSA were strong biofilm producers (value-2-biofilms), while the remaining isolates lacked this ability completely. Summarizing the results of Table 2 and Supplemental Table 3, neither the *agr* type nor the non-wt variants of AgrA or AgrC were attributable to the ability to form a stable, non-removable biofilm on inert polystyrene surfaces.

### AgrA and AgrC variations influence biofilm architecture

**Figure 6:**
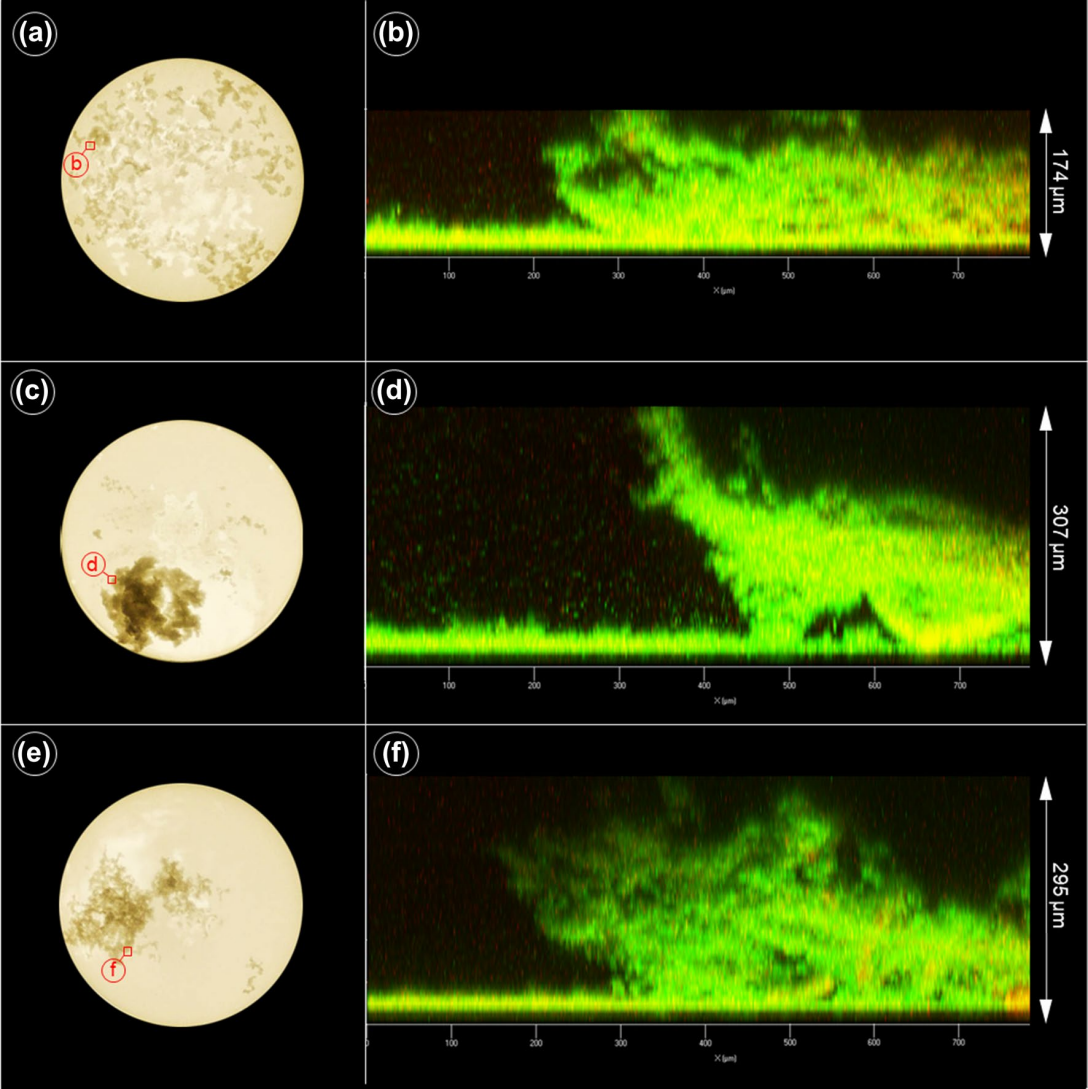
*Agr* activity and biofilm formation differences among *mec*C-MRSA belonging to clonal complex 130. (**a**) Macroscopic camera image of an *S. aureus* isolate (*mec*C-MRSA) harbouring the *agr *III wild type (wt) variant (IMT38119) grown in a 24-well plate and (**b**) the confocal laser scanning picture showing the biofilm profile at the indicated spot (red square). (**c**) Isolate harbouring a *agr *III variant (non-wt *agr*C variant) lacking *agr* activity (RKI5966) and (**d**) its corresponding biofilm. (**e**) Isolate lacking *agr* activity (IMT31819) and (**f**) its corresponding biofilm.

While the crystal violet-staining assay exclusively detects robust biofilms that remain permanently attached to surfaces upon washing, CLSM imaging allows for monitoring the biofilm development in situ*.* By this approach, even delicate interactions of bacterial communities on surfaces can be visualized. In order to elucidate the potential impact of *agr* functionality on biofilm architecture, we selected three representative isolates of the CC130 carrying different genomic variants of *agr* (marked by * in Table 2 and Supplemental Table 4) and analyzed them by CLSM imaging. Although the selected isolates were biofilm-negative in the crystal violet-staining assay on polystyrene, they clearly displayed an in situ biofilm on glass slides during CLSM monitoring, with remarkable differences between the isolates. Thus, the biofilm of the Hld-producing isolate IMT38119 harbouring the CC130-wt *agr* system, formed a flat, dense biofilm that covered almost the entire area of the well (Fig. 6a,b). In contrast, isolate RKI5966 (a non-wt AgrC-variant lacking Hld production) built a dense, local aggregate on the slide (Fig. 6c), with the biofilm mass growing much higher than that of *agr*-WT isolate IMT38119 (i.e. 307 µm vs. 174 µm) (Fig. 6b,d). Similarly, isolate IMT31819, a non-wt AgrA variant lacking Hld production, also formed a local aggregate (Fig. 6e) and displayed a tall biofilm mass whose architecture, however, appeared less dense than those of the other two isolates tested (Fig. 6f). The combined data suggest that the *agr* system may rather influence the biofilm architecture than the overall biofilm-forming capacity of *S. aureus*.

## Discussion

### Agr variants fine-tune virulence levels in *mec*C-MRSA belonging to CC130, CC1943, CC599 and CC49

In the past decade, MRSA have established both as commensals and as infectious agents in animals at alarming rates^87,88^. In addition to classical *mec*A-carrying MRSA clonal lineages, emerging *mec*C-MRSA further add to this problem by affecting various animal species as well as humans, suggesting a broad host range of such strains^8,14,16^. The *mec*C-MRSA isolates analysed in our study were found to belong to clonal complexes CC130, CC1943, CC599 and CC49, confirming the widespread occurrence of these lineages, at least in Europe^8, 14,16^. Each of the four CCs harboured distinct isolates with altered *agr* loci, resulting in reduced or even total loss of *agr* activity. By employing mass spectrometry, we determined Hld production which served as a proxy for RNAIII transcription in our experiments. Agr functionality was further assessed by testing haemolysis on SBA and a colony spreading assay on semisolid agar whose accuracy for predicting Agr activity was shown before^35,71^. Based on the δ-haemolysin amounts detected, the 45 isolates (and three reference strains) were assigned to distinct *agr* activity groups (i.e. 0, +, ++) which matched well with synergistic haemolysis on SBA (Fig. 4a) and the results of the colony spreading assay (Fig. 4b).

Isolates showing amino acid changes in the C-terminal DNA-binding domain of the AgrA response regulator^89^ such as IMT31818 (ΔN177), IMT39824 (F196S), IMT34478 (S215P) and IMT36952 (C199R) did not produce δ-haemolysin, indicating that these aberrations silenced the *agr* system. However, IMT34478 was still capable of colony spreading, suggesting residual or *agr*-independent PSM production by a mechanism that still needs to be established.

With respect to non-wt AgrC variants, RKI5966 (Q16H) showed a variation in the transmembrane 1 domain of the protein, while the aa exchange E216K in IMT32929 is located in the C-terminal dimerization/histidine phosphotransfer subdomain of the protein^90^. These changes are prone to impair AgrC-mediated AIP sensing and signal transduction^90^, and are therefore likely to cause the *agr*-negative phenotype observed in the isolates.

Further, comparative analysis of Hld production values with the CAMP test results confirmed that an active *agr* system is necessary to induce the lunar-shaped synergistic haemolysis on SBA, with Hld and/or PSMs known to contribute to the phenotype^67^. With respect to β-haemolysin production, *agr* was shown to have a major impact on *hlb* transcription^91^. However, this association is obviously not straightforward and applicable for all strains. Thus, high levels of Hlb were also detected in *S. aureus* isolates displaying low *agr* activity, such as RN4220^86^, which is in good agreement with the Hlb detection results obtained in our isolates (Supplemental Figure 2). Moreover, our data indicate that at least a weak *agr* activity is necessary to induce a Hlb-mediated β-haemolysis phenotype on SBA, even when the intracellular Hlb levels were apparently sufficient to induce haemolysis in corresponding *agr* wt-isolates (Supplemental Figure 2).

Production of many secreted enzymes involved in lipid and protein degradation and haemolysins are influenced or even directly controlled by *agr*. The main effector of this locus, RNAIII, is known to promote α-haemolysin expression on the transcriptional and post-transcriptional level^92^. As expected, Hla production was not detected in isolates lacking the gene (IMT34489) and in those carrying *hla* frameshift mutations (IMT34479 and IMT34480). Surprisingly, Hla abundance values measured in whole cells of isolates with intact *hla* genes did not correlate with the *agr* activity levels (Supplemental Figure 1). Also, for some of the isolates with AgrA/AgrC aberrations and negative α-haemolysis on SBA (IMT31818, IMT32510, IMT32929, IMT36945, IMT39824, RKI5966, NE1532, and RN4220) we noticed intracellular Hla protein amounts that were comparable to that of *agr*-wt strains showing a haemolytic phenotype (Supplemental Figure 1). A study by Montgomery et al. confirmed notable *agr*-independent transcription of Hla in an *agr*-deficient strain (USA300 lineage), while the protein was not detected in the corresponding culture supernants^69^, which is commonly explained by the promoting role of RNAIII for *hla* transcript translation^92^. Since our Hlb and Hla protein abundances were measured from overnight-grown cells after washing with PBS, our results indicate that some *agr*-activity is needed for toxin release. Interestingly, extracellular vesicles released from cells of the USA300 lineage were found to contain Hla^93^. In that particular study, PSMα was identified to promote biogenesis of extracellular vesicles filled with proteins by destabilisation of the cytoplasmic membrane^93^. Thus, is tempting to speculate that baseline *agr*-independent *hla* transcription and translation might occur in the variants, leading to intracellular accumulation of α-haemolysin. Lacking PSM activity (due to AgrA/C variations) might prevent release of Hla in the supernatant, resulting in the haemolysis-negative phenotypes observed. More experimental work, however, is needed to substantiate this hypothesis in the future.

### Agr variants influence biofilm structure and density

Biofilm formation is a key factor in pathogenesis of persistent staphylococcal infections^94^, and downregulation of *agr* is supposed to facilitate biofilm development in staphylococci^50,95–97^ with strain-specific differences occurring particularly among clinical isolates^98^. When testing the *mec*C-MRSA isolates in a standard crystal violet-staining biofilm assay, we did not find an association between *agr* functionality and stable biofilm formation in polystyrene tissue culture plates (Table 2), which is in good agreement with previous findings in clinical MRSA clonal lineages^38^. Generally, we found only a few biofilm-forming isolates by this assay, although biofilm-associated genes were present and intact in all strains. In comparison to *S. epidermidis*, *S. aureus* is known to show a much weaker biofilm detection performance in the standard biofilm assay. Rather than suggesting a lower overall biofilm-forming capacity of *S. aureus*, the phenomenon may reflect a general mechanically instable contact of biofilm-associated *S. aureus* cells to inert surfaces, leading to removal of loosely attached biofilm structures upon washing of the plates. Indeed, when using CLSM imaging, which does not involve washing of the cover slips, we detected visible biofilms, at least in the three CC130 isolates analysed (Fig. 6). Interestingly, strains possessing different *agr* activity levels displayed differences regarding biofilm thickness and architecture. Thus, while the WT-*agr* isolate formed a well-organized biofilm (Fig. 6b), the two *agr*-deficient variants displayed a much higher biofilm mass which, however, appeared less-structured (Fig. 6d,f). This might be due to the lack of *agr*-dependent production of PSMs which were previously shown to play an eminent role in functional biofilm architecture^99,100^. *S. aureus* biofilm formation is a complex and highly dynamic process which comprises, according to a recently newly defined five-stage model, attachment, multiplication, exodus, maturation and dispersal of the biofilm^101^. Apart from its function in maturation and dispersal (involving *agr*-controlled proteases and PSMs), *agr* also plays a significant role during initial attachment by facilitating (in the early growth stage) expression of cell wall-anchored proteins that mediate host matrix protein binding as well as contact to abiotic surfaces^102^. We currently speculate that the loose biofilm structure in the CC130 *agr*-variants might be associated with a diminished initial attachment of the bacteria to the surface and/or to each other. However, it is conceivable that other processes known to shape the biofilm architecture such as programmed autolysis and eDNA release^103^ might be (indirectly) affected by the Agr variations as well. But, clearly more experimental work is needed to substantiate this hypothesis.

### Adaptation strategies of *mec*C-MRSA might involve *agr* defectiveness and carriage of *agr*-independent virulence factors

Evolution and changes of bacterial virulence is highly dynamic and difficult to predict^104,105^. Attenuated virulence favouring host colonization and/or persistence of infection (e.g. small colony variants) is a common concept in bacterial evolution^106–108^. The *mec*C-MRSA lineages CC130, CC49, CC1943 and CC599 were obviously not among those CC’s frequently reported for nasal colonization in humans and animals such as CC22 and CC398^109–112^. Consequently, other strategies might promote their viability or even spread among mammalian hosts and the environment. On one hand, the disability to produce the *agr*D-encoded auto-inducer peptide (AIP) may prevent costly competition of invading strains with an “incompatible” *agr* system harboured by other *S. aureus* lineages or even other staphylococcal species in a particular host^113^. One the other hand, presence of a compatible AIP system allows the cells to take advantage of the *agr-*induced factors produced by co-habiting staphylococci^29^. A highly instructive study, using a wax moth larva virulence model, revealed that a functional *agr* system is necessary for a cooperative and beneficial “behaviour” of the local population, while *agr* defective mutants exploit (“cheat”) on the cells with a functional *agr* system, allowing them to prevail when grown in mixed populations with cooperators^113^. Based on earlier published results of growth competition experiments^113, 114^, an increased viability of *agr* defective *S. aureus* mutants among resident staphylococci of different host species seems to be likely. This view is further supported by a recent study, showing that distinct external stress conditions drive the selection of Agr quorum-sensing mutants that may confer a fitness advantage to the *S. aureus* population^115^.

Unspecific and unregulated T-cell stimulators (superantigens) such as enterotoxins, enterotoxin-like proteins, and the toxic shock syndrome toxin contribute to host cell damages inducible by *S. aureus*. Combinations of genes encoding these superantigens were identified in varying frequencies for all four lineages reported on here (Fig. 1). With respect to enterotoxins^116^, Enterotoxin C (SEC), which is commonly located on a SaPI (reviewed in^49^), was identified in isolates belonging to CC130, CC599 and CC1943. However, SEC production requires *agr*-depending *rot* degradation^117^, which is defective in some of the isolates reported on here. While classical enterotoxins such as SEA-C are regulated by the *agr* system, the “novel” enterotoxins and variants of the toxic shock syndrome toxin seem to be *agr* independent (reviewed in^49^). In line with this, we have shown expression of the protein encoded by *tst-*bov in isolates showing lacking detectable *agr* activity (Fig. 1, Table 2), exemplified by isolates IMT31818 and IMT39824. The variants of tst-*bov* were harboured by *S. aureus* pathogenicity islands showing mosaic structures of known and novel SaPIs, an observation which has been reported (e.g. for human clinical isolates) before^118^.

## Conclusion

Comparative genomics of the *agr* encoding region allow identification of variants deviating from the wildtype in *mec*C-MRSA belonging to CC130, CC599, CC49 and CC1943, and subsequent proteomics revealed the capability of each altered *agr* system to transcribe RNAIII, which was directly mirrored by the corresponding Hld protein values. Our research indicates that *mec*C-MRSA with *agr* variations are defective for *agr*-depending quorum sensing, harbour additional *agr*-independent virulence factors and exhibit varying biofilm properties as a likely part of their survival strategy. In bacteria, adaptation to a changing environment is often associated with the selection for mutations in (virulence factor) genes that become dispensable or disadvantageous in the novel niche (reviewed in^104^). This concept can obviously be extended to global regulators such as the *agr* quorum-sensing system as well, resulting in pleiotropic effects and the generation of phenotypic heterogeneity that might further support the establishment of emerging clonal lineages in new hosts and niches.

## Supplementary information

Supplementary Information.

## Data Availability

Genomic sequencing data used are available for download from the National Center for Biotechnology Information (NCBI) under BioProject accessions PRJNA588740. Accession numbers of whole genomes sequences are provided in Supplemental Table 2. The mass spectrometry proteomics data have been deposited to the ProteomeXchange Consortium (https://proteomecentral.proteomexchange.org) via the PRIDE partner repository with the dataset identifier PXD016486.
