# Supplementary material for: Silence as a way of niche adaptation: mecC-MRSA with variations in the accessory gene regulator (agr) functionality express kaleidoscopic phenotypes
Source: Sci Rep. 2020 Sep 8;10:14787. doi: 10.1038/s41598-020-71640-4 (PMC7479134; doi:10.1038/s41598-020-71640-4)
Supplement: Supplementary file 1 — Supplementary Information. [file 41598_2020_71640_MOESM1_ESM.pdf]

Silence as a way of niche adaptation: *mec*C-MRSA with variations in the accessory gene regulator (*agr*) functionality express kaleidoscopic phenotypes

Charlotte Huber[1], Ivonne Stamm[2], Wilma Ziebuhr[3], Gabriella Marincola[3], Markus Bischoff[4], Birgit Strommenger[5], Greta Jaschkowitz[3], Tessa Marciniak[3], Christiane Cuny[5], Wolfgang Witte[5], Joerg Doellinger[6], Christoph Schaudinn[1], Andrea Thürmer[7], Lennard Epping[8], Torsten Semmler[8], Antina Lübke-Becker[9], Lothar H. Wieler[10] and Birgit Walther[1*]

[1]Advanced Light and Electron Microscopy (ZBS4), Robert Koch Institute, Berlin, Germany

[2]IDEXX GmbH, Ludwigsburg, Germany

[3]Institute for Molecular Infection Biology, University of Würzburg, Würzburg, Germany

[4]Institute of Medical Microbiology and Hygiene, Saarland University, Homburg, Germany

[5]National Reference Centre for Staphylococci and Enterococci, Robert Koch Institute, Wernigerode, Germany

[6]Proteomics and Spectroscopy (ZBS6), Robert Koch Institute, Berlin, Germany

[7]Genome Sequencing (MF2), Robert Koch Institute, Berlin, Germany

[8]Microbial Genomics (NG1), Robert Koch Institute, Berlin, Germany

[9]Institute of Microbiology and Epizootics, Freie Universität Berlin, Berlin, Germany

[10]Methodology and Research Infrastructure, Robert Koch Institute, Berlin, Germany

*corresponding author: waltherb@rki.de

Supplemental figure 1. Synergistic production of different haemolysins and alpha haemolysin (Hla) protein abundance levels

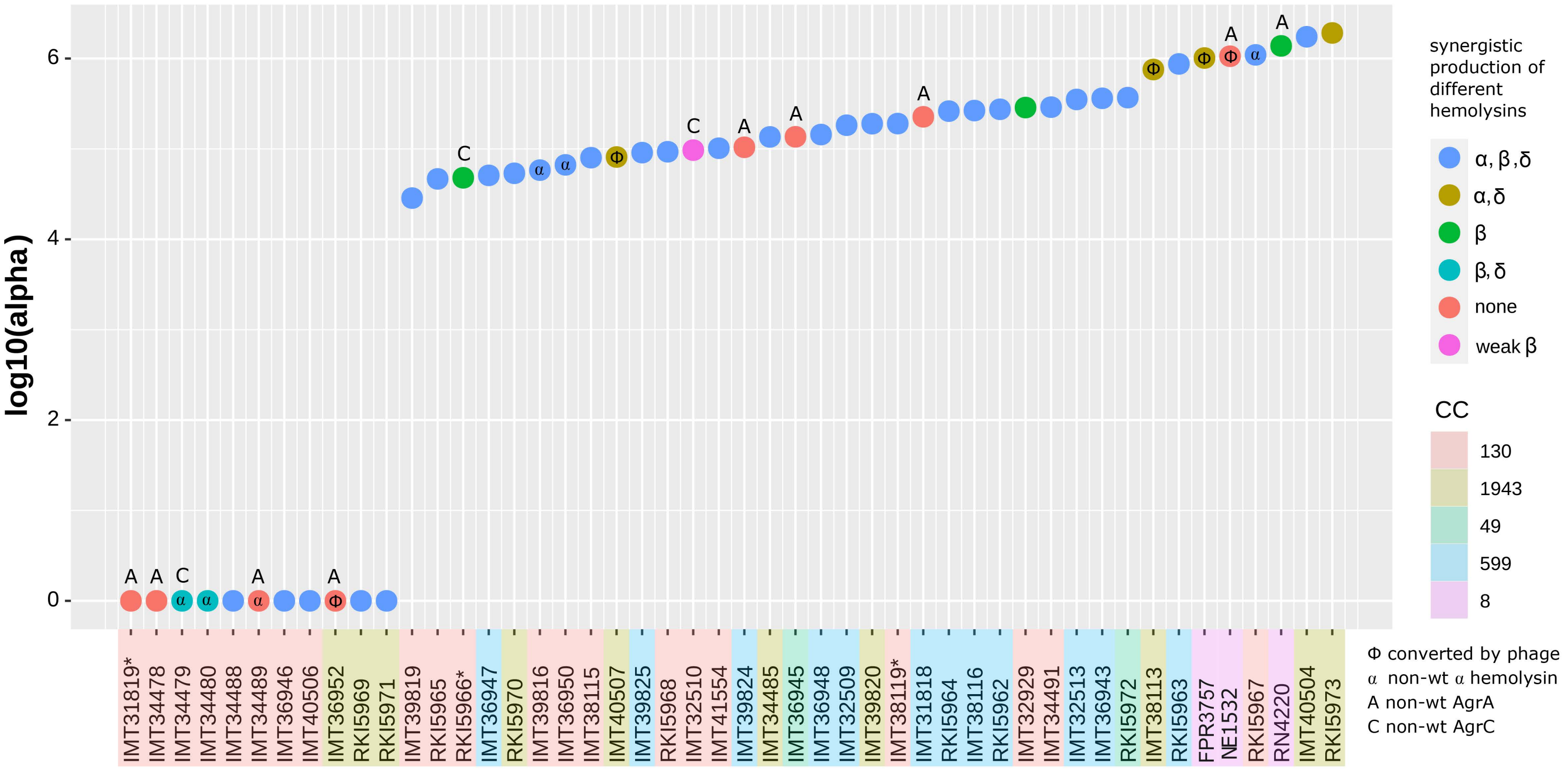

Supplemental figure 2. CAMP phenomenon and β-haemolysin (Hlb) protein abundances

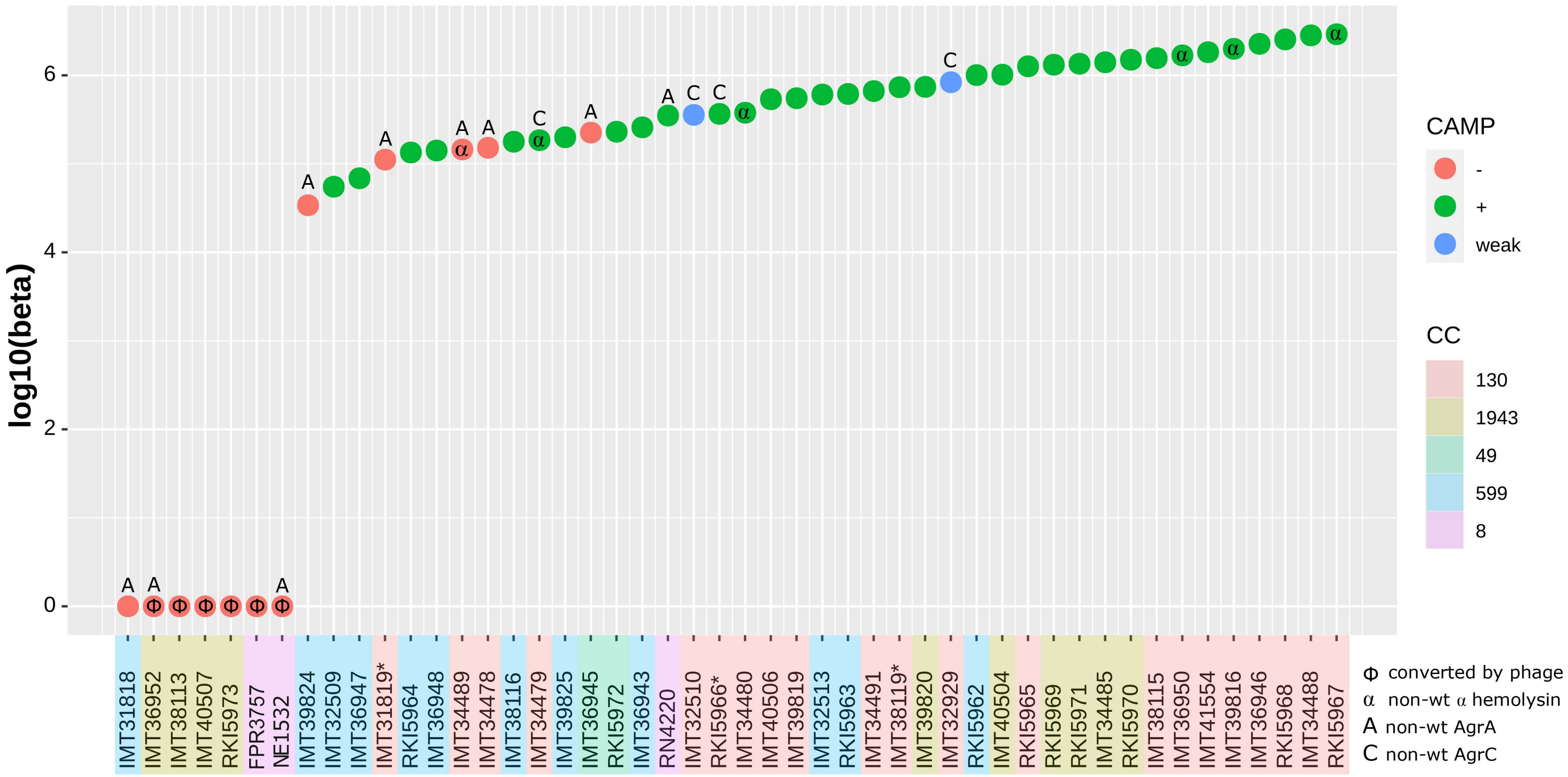

Supplementary Information file

Supplemental Table 1: Dynamic window sizing for mass spectrometry

**Mass spectrometry**		
start [m/z]	end [m/z]	width [m/z]
349,5	382,5	33
381,5	406,5	25
405,5	422,5	17
421,5	437,5	16
436,5	449,5	13
448,5	460,5	12
459,5	469,5	10
468,5	478,5	10
477,5	487,5	10
486,5	496,5	10
495,5	505,5	10
504,5	514,5	10
513,5	522,5	9
521,5	531,5	10
530,5	540,5	10
539,5	548,5	9
547,5	555,5	8
554,5	563,5	9
562,5	572,5	10
571,5	581,5	10
580,5	590,5	10
589,5	599,5	10
598,5	608,5	10
607,5	617,5	10
616,5	626,5	10
625,5	635,5	10
634,5	644,5	10
643,5	652,5	9
651,5	661,5	10
660,5	671,5	11
670,5	680,5	10
679,5	690,5	11
689,5	700,5	11
699,5	710,5	11
709,5	721,5	12
720,5	733,5	13
732,5	745,5	13
744,5	758,5	14

757,5	770,5	13
769,5	782,5	13
781,5	795,5	14
794,5	811	16
810	827	17
825,5	843,5	18
842,5	860,5	18
859,5	880,5	21
879,5	902,5	23
901,5	926,5	25
925,5	954,5	29
953,5	986,5	33
985,5	1023,5	38
1022,5	1070,5	48
1069,5	1150,5	81

Supplemental Table 2: Genome Accession Numbers

BioProject accessions PRJNA588740

**Genome accession numbers**	
**sample-ID**	**accession**
IMT31818	SRR10426558
IMT31819	SRR10426557
IMT32509	SRR10426546
IMT32510	SRR10426535
IMT32513	SRR10426524
IMT32929	SRR10426518
IMT34478	SRR10426517
IMT34479	SRR10426516
IMT34480	SRR10426515
IMT34485	SRR10426514
IMT34488	SRR10426556
IMT34489	SRR10426555
IMT34491	SRR10426554
IMT36943	SRR10426553
IMT36945	SRR10426552
IMT36946	SRR10426551
IMT36947	SRR10426550
IMT36948	SRR10426549
IMT36950	SRR10426548
IMT36952	SRR10426547
IMT38113	SRR10426545

IMT38115	SRR10426544
IMT38116	SRR10426543
IMT38119	SRR10426542
IMT39816	SRR10426541
IMT39819	SRR10426540
IMT39820	SRR10426539
IMT39824	SRR10426538
IMT39825	SRR10426537
IMT40504	SRR10426536
IMT40506	SRR10426534
IMT40507	SRR10426533
IMT41554	SRR10426532
RKI5962	SRR10426531
RKI5963	SRR10426530
RKI5964	SRR10426529
RKI5965	SRR10426528
RKI5966	SRR10426527
RKI5967	SRR10426526
RKI5968	SRR10426525
RKI5969	SRR10426523
RKI5970	SRR10426522
RKI5971	SRR10426521
RKI5972	SRR10426520
RKI5973	SRR10426519

Supplemental Table 3:). Pairwise SNP-distances between the core genomes were calculated for all MRSA isolates

IMT32929	IMT34488	RKI5965	IMT39816	IMT39819	RKI5966	IMT34489	IMT34478	IMT31819	IMT40506	IMT39825	RKI5962	RKI5964	RKI5963	IMT36948	IMT36947	IMT32513	IMT39824	IMT36943	IMT31818	IMT38116	IMT32509
15161	15158	15161	15172	15137	15133	15151	15141	15138	15140	15341	15331	15374	15411	15385	15385	15330	15346	15326	15343	15354	15334
15130	15127	15130	15141	15106	15102	15120	15110	15107	15109	15312	15302	15345	15382	15356	15356	15301	15317	15297	15314	15325	15305
10509	10506	10509	10514	10485	10476	10495	10487	10482	10484	10934	10922	10965	11006	10976	10976	10923	10935	10917	10936	10947	10927
10502	10497	10502	10507	10478	10469	10488	10480	10475	10477	10927	10915	10958	10999	10969	10969	10916	10928	10910	10929	10940	10920
10521	10518	10521	10526	10497	10488	10507	10499	10494	10496	10946	10934	10977	11018	10988	10988	10935	10947	10929	10948	10959	10939
10514	10511	10514	10519	10490	10481	10500	10492	10487	10489	10940	10928	10971	11012	10982	10982	10929	10941	10923	10942	10953	10933
10512	10509	10512	10517	10488	10479	10498	10490	10485	10487	10939	10927	10970	11011	10981	10981	10928	10940	10922	10941	10952	10932
10502	10499	10502	10507	10478	10469	10488	10480	10475	10477	10929	10917	10960	11001	10971	10971	10918	10930	10912	10931	10942	10922
10511	10508	10511	10516	10487	10478	10497	10489	10484	10486	10937	10925	10968	11009	10979	10979	10926	10938	10920	10939	10950	10930
10511	10508	10511	10516	10487	10478	10497	10489	10484	10486	10937	10925	10968	11009	10979	10979	10926	10938	10920	10939	10950	10930
10510	10507	10510	10515	10486	10477	10496	10488	10483	10485	10936	10924	10967	11008	10978	10978	10925	10937	10919	10938	10949	10929
10548	10545	10550	10555	10526	10517	10536	10528	10523	10525	10974	10962	11005	11046	11016	11016	10963	10975	10957	10976	10987	10967
349	346	347	354	323	315	335	329	322	324	10008	9995	10039	10081	10050	10050	9993	10009	9991	10010	10021	10001
354	351	354	361	332	322	342	336	329	331	10015	10002	10046	10088	10057	10057	10000	10016	9998	10017	10028	10008
310	307	308	315	286	276	296	290	283	285	9971	9958	10002	10044	10013	10013	9956	9972	9954	9973	9984	9964
405	402	403	409	381	371	391	385	378	380	10057	10044	10088	10129	10099	10099	10042	10058	10040	10059	10070	10050
225	222	297	304	275	265	285	279	272	274	9976	9963	10007	10049	10018	10018	9961	9977	9959	9976	9987	9967
250	247	322	329	300	290	310	304	297	299	9998	9985	10029	10071	10040	10040	9983	9999	9981	9998	10009	9989
204	201	282	289	260	250	270	264	257	259	9964	9951	9995	10037	10006	10006	9949	9965	9947	9964	9975	9955
229	226	317	324	295	285	305	299	292	294	9994	9981	10025	10067	10036	10036	9979	9995	9977	9994	10005	9985
234	231	321	328	300	290	308	304	297	299	10003	9990	10034	10076	10045	10045	9988	10004	9986	10003	10014	9994
237	234	324	331	303	293	311	307	300	302	10006	9993	10037	10079	10048	10048	9991	10007	9989	10006	10017	9997
238	235	334	341	312	302	322	316	309	311	10009	9996	10040	10082	10051	10051	9994	10010	9992	10009	10020	10000
	145	342	349	320	310	330	324	317	319	10019	10006	10050	10092	10061	10061	10004	10020	10002	10019	10030	10010
145		338	346	317	307	327	321	314	316	10016	10003	10047	10089	10058	10058	10001	10017	9999	10016	10027	10007
342	338		183	204	200	220	214	207	209	10017	10004	10048	10090	10059	10059	10002	10018	10000	10017	10028	10008
349	346	183		211	207	227	221	214	216	10022	10009	10053	10095	10064	10064	10007	10023	10005	10022	10033	10013
320	317	204	211		178	198	192	185	187	9994	9981	10025	10067	10036	10036	9979	9995	9977	9994	10005	9985
310	307	200	207	178		178	172	165	167	9987	9974	10018	10060	10029	10029	9972	9988	9970	9987	9998	9978
330	327	220	227	198	178		94	101	103	10005	9992	10036	10078	10047	10047	9990	10006	9988	10005	10016	9996
324	321	214	221	192	172	94		95	97	9997	9984	10028	10070	10039	10039	9982	9998	9980	9997	10008	9988
317	314	207	214	185	165	101	95		40	9994	9981	10025	10067	10036	10036	9979	9995	9977	9994	10005	9985
319	316	209	216	187	167	103	97	40		9996	9983	10027	10069	10038	10038	9981	9997	9979	9996	10007	9987
10019	10016	10017	10022	9994	9987	10005	9997	9994	9996		74	221	269	234	234	171	185	165	186	197	177
10006	10003	10004	10009	9981	9974	9992	9984	9981	9983	74		209	257	222	222	159	173	153	174	185	165
10050	10047	10048	10053	10025	10018	10036	10028	10025	10027	221	209		302	267	267	210	224	204	225	236	216
10092	10089	10090	10095	10067	10060	10078	10070	10067	10069	269	257	302		267	267	258	272	252	273	284	264
10061	10058	10059	10064	10036	10029	10047	10039	10036	10038	234	222	267	267		0	223	237	217	238	249	229
10061	10058	10059	10064	10036	10029	10047	10039	10036	10038	234	222	267	267	0		223	237	217	238	249	229
10004	10001	10002	10007	9979	9972	9990	9982	9979	9981	171	159	210	258	223	223		124	122	143	154	134
10020	10017	10018	10023	9995	9988	10006	9998	9995	9997	185	173	224	272	237	237	124		136	157	168	148
10002	9999	10000	10005	9977	9970	9988	9980	9977	9979	165	153	204	252	217	217	122	136		133	144	124
10019	10016	10017	10022	9994	9987	10005	9997	9994	9996	186	174	225	273	238	238	143	157	133		57	37
10030	10027	10028	10033	10005	9998	10016	10008	10005	10007	197	185	236	284	249	249	154	168	144	57		46
10010	10007	10008	10013	9985	9978	9996	9988	9985	9987	177	165	216	264	229	229	134	148	124	37	46	

Supplemental table 4: Amino acid variation in the agr system of *mec*C MRSA

	Amino acid sequences (predicted)								
		Hemolysins				Accessory gene regulatory system			
ID	CC	alpha	beta	delta	*agr*	AgrA	AgrB	AgrC	AgrD
IMT31818	599	WT	WT	WT	I	ΔN177	A182T	WT	WT
IMT32509	599	WT	WT	WT	I	WT	A182T	WT	WT
IMT32513	599	WT	WT	WT	I	WT	A182T	WT	WT
IMT36943	599	WT	WT	WT	I	WT	A182T	WT	WT
IMT36947	599	WT	WT	WT	I	WT	A182T	WT	WT
IMT36948	599	WT	WT	WT	I	WT	A182T	WT	WT
IMT38116	599	WT	WT	WT	I	WT	A182T	WT	WT
IMT39824	599	WT	WT	WT	I	F196S	A182T	WT	WT
IMT39825	599	WT	WT	WT	I	WT	A182T	WT	WT
RKI5962	599	WT	WT	WT	I	WT	A182T	WT	WT
RKI5963	599	WT	WT	WT	I	WT	A182T	WT	WT
RKI5964	599	WT	WT	WT	I	WT	A182T	WT	WT
IMT36945	49	WT	WT	WT	II	short	Y76C	WT	WT
RKI5972	49	WT	WT	WT	II	WT	Y76C	WT	WT
IMT31819*	130	WT	WT	WT	III	IS	WT	WT	WT
IMT32510	130	WT	WT	WT	III	WT	WT	G284D	WT
IMT32929	130	WT	WT	WT	III	WT	WT	E216K	WT
IMT34478	130	WT	WT	WT	III	S215P	WT	WT	WT
IMT34479	130	α IS	WT	WT	III	WT	WT	N5S	WT
IMT34480	130	α IS	WT	WT	III	WT	WT	WT	WT
IMT34488	130	WT	WT	WT	III	WT	WT	WT	WT
IMT34489	130	Δ	WT	WT	III	G68D	WT	WT	WT
IMT34491	130	WT	WT	WT	III	WT	WT	WT	WT

IMT36946	130	WT	WT	WT	III	WT	WT	WT	WT
IMT36950	130	T12I	WT	WT	III	WT	WT	WT	WT
IMT38115	130	WT	WT	WT	III	WT	WT	WT	WT
IMT38119*	130	WT	WT	WT	III	WT	WT	WT	WT
IMT39816	130	K189E	WT	WT	III	WT	WT	WT	WT
IMT39819	130	WT	WT	WT	III	WT	WT	WT	WT
IMT40506	130	WT	WT	WT	III	WT	WT	WT	WT
IMT41554	130	WT	WT	WT	III	WT	WT	WT	WT
RKI5965	130	WT	WT	WT	III	WT	WT	WT	WT
RKI5966*	130	WT	WT	WT	III	WT	WT	Q16H	WT
RKI5967	130	D211G	WT	WT	III	WT	WT	WT	WT
RKI5968	130	WT	WT	WT	III	WT	WT	WT	WT
IMT34485	1943	WT	WT	WT	IV	WT	WT	I64S	WT
IMT36952	1943	WT	P	WT	IV	C199R	WT	I64S	WT
IMT38113	1943	WT	P	WT	IV	WT	WT	I64S	WT
IMT39820	1943	WT	WT	WT	IV	WT	WT	I64S	WT
IMT40504	1943	WT	A68V	WT	IV	WT	WT	I64S	WT
IMT40507	1943	WT	P	WT	IV	WT	WT	I64S	WT
RKI5969	1943	WT	WT	WT	IV	WT	WT	I64S	WT
RKI5970	1943	WT	WT	WT	IV	WT	WT	I64S	WT
RKI5971	1943	WT	WT	WT	IV	WT	WT	I64S	WT
RKI5973	1943	WT	P	WT	IV	WT	WT	I64S	WT

Amino acid sequences for alpha, beta and delta haemolysins and the *agr* system of 45 *mec*C-MRSA. Position of amino acid changes with respect to the wild type (WT) reference sequence (below) are shown. Abbreviations:
ID, isolate number; CC, clonal complex; *agr*, accessory gene regulatory type; Δ, deletion; IS, insertion; *, isolates further investigated with respect to biofilm structures using CLSM.

NCBI Reference sequences (aa) and strain IDs									
		AGR I		AGR II		AGR III		AGR IV	
		accession	strain	accession	strain	accession	strain	accession	strain
Agr	AgrA	P0A0I6.1	MW2	P0A0I6.1	MW2	P0A0I6.1	MW2	P0A0I6.1	MW2
	AgrB	QBY49017.1	O268	CZQ66246.1	1943STDY5698362	AUG74347.1	O46	SAO20788.1	n.a.
	AgrC	EVG59461.1	OCMM6067	KIT97067.1	SA-120	KIT81064.1	SA-006 (N5S)	ABB17521.1	H560
	AgrD	YP_500744.1	NCTC8325	ABQ49857.1	JH9	EFH25310.1	ATCC 51811	AGU55642.1	6850
Hemolysins	alpha	EFK81723.1	TCH70						
	beta	AAB32218.1	126/89						
	delta	Q2FWM8	NCTC8325						
Abbreviations: n.a. not available; accession, NCBI accession number for amino acid sequence; strain, wild type/reference strain									

Supplemental table 5. Amino acid variations in global regulators (Protein ID / Strain ID)

	SaeS	SaeR	SrrA	SrrB	ArlR	ArlS	SarA	SarR	Rot	MgrA	SigB
	Histidine protein kinase	Response regulator	Transcrip-tional regulatory protein	Sensor protein	Response regulator	Signal transduc-tion histidine-protein kinase	Transcrip-tional regulator	HTH-type transcrip-tional regulator	Repressor of toxins	HTH-type transcrip-tional regulator	RNA poly-merase sigma factor
**Reference**	Q7A6V4 / N315	Q7A6V3 / N315	Q7A5H6 / N315	Q8NWF3 / MW2	Q9KJN4 / NCTC 8325	WP_06413 3950.1	Q7A732 / N315	Q2FEJ8-1 / USA300	EEW4745 2.1	Q7A6X2 / N315	P95844
IMT31818	WT	WT	WT	A322T; V434I (CC599)	WT	WT	WT	WT	WT	WT	WT
IMT32509	WT	WT	WT	A322T; V434I (CC599)	WT	WT	WT	WT	WT	WT	WT
IMT32513	WT	WT	WT	A322T; V434I (CC599)	WT	WT	WT	WT	WT	WT	WT
IMT36943	WT	WT	WT	A322T; V434I (CC599)	WT	WT	WT	WT	WT	WT	WT
IMT36947	WT	WT	WT	A322T; V434I (CC599)	WT	WT	WT	WT	WT	WT	WT
IMT36948	WT	WT	WT	A322T; V434I (CC599)	WT	WT	WT	WT	WT	WT	WT
IMT38116	WT	WT	WT	A322T; V434I (CC599)	WT	WT	WT	WT	WT	WT	WT
IMT39824	WT	WT	WT	A322T; V434I (CC599)	WT	WT	WT	WT	WT	WT	WT
IMT39825	WT	WT	WT	A322T;	WT	WT	WT	WT	WT	WT	WT

				V434I (CC599)							
RKI5962	WT	WT	WT	A322T; V434I (CC599)	WT	WT	WT	WT	WT	WT	WT
RKI5963	WT	WT	WT	A322T; V434I (CC599)	WT	WT	WT	WT	WT	WT	WT
RKI5964	WT	WT	WT	A322T; V434I (CC599)	WT	WT	WT	WT	WT	WT	WT
**Reference**	Q7A6V4 / N315	Q7A6V3 / N315	Q7A5H6 / N315	Q8NWF3 / MW2	Q9KJN4 / NCTC 8325	WP_064133950.1	Q7A732 / N315	Q2FEJ8-1 / USA300	EEW47452.1	Q7A6X2 / N315	P95844
IMT36945	WT	WT	WT	V434I; D509A	WT	WT	WT	WT	WT	WT	WT
RKI5972	WT	WT	WT	V434I; D509A	WT	WT	WT	WT	WT	WT	WT
**Reference**	Q7A6V4 / N315	Q7A6V3 / N315	Q7A5H6 / N315	Q8NWF3 / MW2	Q9KJN4 / NCTC 8325	WP_064133950.1	Q7A732 / N315	Q2FEJ8-1 / USA300	EEW47452.1	Q7A6X2 / N315	P95844
IMT31819	WT	WT	WT	WT	WT	WT	WT	WT	WT	WT	WT
IMT32510	WT	E225G	WT	WT	WT	WT	WT	WT	WT	WT	WT
IMT32929	WT	WT	WT	WT	WT	WT	WT	WT	WT	WT	A253V
IMT34478	WT	WT	WT	WT	WT	WT	WT	WT	WT	WT	WT
IMT34479	WT	WT	WT	WT	WT	WT	WT	WT	WT	WT	WT
IMT34480	WT	WT	WT	WT	WT	WT	WT	WT	WT	WT	WT
IMT34488	WT	WT	WT	WT	WT	WT	WT	WT	WT	WT	WT
IMT34489	WT	WT	WT	WT	WT	WT	WT	WT	WT	WT	WT
IMT34491	WT	WT	WT	WT	WT	WT	WT	WT	WT	WT	WT
IMT36946	WT	WT	WT	WT	WT	WT	WT	WT	WT	WT	WT
IMT36950	WT	WT	WT	WT	WT	WT	WT	WT	WT	WT	WT
IMT38115	WT	WT	WT	WT	WT	WT	WT	WT	WT	WT	WT
IMT38119	WT	WT	WT	T123S	WT	WT	WT	WT	R59I	WT	WT
IMT39816	WT	WT	WT	WT	WT	WT	WT	WT	WT	WT	WT
IMT39819	WT	WT	WT	WT	WT	WT	WT	WT	WT	WT	WT
IMT40506	WT	WT	WT	WT	WT	WT	WT	WT	WT	WT	WT
IMT41554	WT	WT	WT	insertion	WT	WT	WT	WT	WT	WT	WT

RKI5965	WT	WT	WT	WT	WT	WT	WT	WT	WT	WT	WT
RKI5966	WT	WT	WT	WT	WT	WT	WT	WT	WT	WT	WT
RKI5967	WT	WT	WT	WT	WT	WT	WT	WT	WT	WT	WT
RKI5968	WT	WT	WT	WT	WT	WT	WT	WT	WT	WT	WT
Reference	Q7A6V4 / N315	Q7A6V3 / N315	Q7A5H6 / N315	Q8NWF3 / MW2	Q9KJN4 / NCTC 8325	WP_10320 5040.1	Q7A732 / N315	Q2FEJ8-1 / USA300	EEW4745 2.1	Q7A6X2 / N315	P95844
IMT34485	WT	WT	WT	WT	WT	WT	WT	WT	WT	WT	WT
IMT36952	WT	WT	WT	WT	WT	WT	WT	WT	WT	WT	WT
IMT38113	D269G	WT	WT	WT	WT	WT	WT	WT	WT	WT	WT
IMT39820	WT	WT	WT	WT	WT	WT	WT	WT	WT	WT	WT
IMT40504	P193L	WT	WT	WT	WT	WT	WT	WT	WT	WT	WT
IMT40507	WT	WT	WT	WT	WT	WT	WT	WT	WT	WT	WT
RKI5969	WT	WT	WT	WT	WT	WT	WT	WT	WT	WT	WT
RKI5970	WT	WT	WT	WT	WT	WT	WT	WT	WT	WT	WT
RKI5971	WT	WT	WT	WT	WT	WT	WT	WT	WT	WT	WT
RKI5973	WT	WT	WT	WT	WT	WT	WT	WT	WT	WT	I214N

WT, wild type

Supplemental table 6: alpha-, beta- and delta- haemolysin abundance levels

	**Haemolysin**		
**ID**	**alpha**	**beta**	**delta**
IMT31818	226466	0	0
IMT32509	182889	55119	40203500
IMT32513	352463	606824	49393600
IMT36943	362910	258289	5991790
IMT36947	50962	68713	25962700
IMT36948	144179	141258	17740600
IMT38116	265550	177798	56901700

IMT39824	104197	34039	0
IMT39825	90945	199562	39853100
RKI5962	274323	1006250	42899800
RKI5963	871106	615917	20333500
RKI5964	261854	134389	29062200
IMT36945	136132	225265	0
RKI5972	369213	230575	64359000
IMT31819*	0	111460	0
IMT32510	96885	357332	242092
IMT32929	286669	835736	167153
IMT34478	0	151672	0
IMT34479	0	184999	11537100
IMT34480	0	377950	11540400
IMT34488	0	2829180	10020100
IMT34489	0	145289	0
IMT34491	289696	663535	13129800
IMT36946	0	2268950	17193500
IMT36950	66586	1679970	16183400
IMT38115	79961	1564050	16393100
IMT38119*	190628	734137	16701500
IMT39816	58233	1993900	15230400
IMT39819	28569	551468	11417000
IMT40506	0	536742	9470570
IMT41554	101750	1824170	12473100
RKI5965	46568	1264390	20195000
RKI5966*	47915	366644	0
RKI5967	1097210	2914410	15388400
RKI5968	93002	2544930	16805900
IMT34485	135750	1403930	69541000

IMT36952	0	0	0
IMT38113	755597	0	67475600
IMT39820	190022	740548	94823900
IMT40504	1740330	1017250	84429200
IMT40507	81362	0	80901100
RKI5969	0	1317800	28418500
RKI5970	53724	1498920	25153000
RKI5971	0	1351630	28530200
RKI5973	1917410	0	37149400
RN4220	1379010	350906	42317
FPR3757	1012840	0	15792500
NE1532	1060150	0	0

* selected for CLSM

Supplemental table 6. Original data from the biofilm crystal violet-staining assay

Biofilm crystal violet assay (original data)

ID	1st biological replicate				2nd biological replicate				3rd biological replicate				mean absorbance 492nm
	**1**	**2**	**3**	**4**	**5**	**6**	**7**	**8**	**9**	**10**	**11**	**12**	
IMT31818	0,29	0,35	0,25	0,26	0,21	0,26	0,21	0,19	0,17	0,25	0,22	0,18	0,2369
IMT31819	0,14	0,15	0,13	0,16	0,19	0,24	0,18	0,16	0,16	0,16	0,14	0,19	0,1647
IMT32509	0,11	0,11	0,12	0,11	0,11	0,12	0,11	0,11	0,12	0,11	0,10	0,12	0,1115
IMT32510	0,21	0,23	0,29	0,20	0,22	0,19	0,20	0,22	0,21	0,33	0,22	0,20	0,2275
IMT32513	0,16	0,22	0,27	0,21	0,32	0,29	0,26	0,28	0,22	0,25	0,20	0,23	0,2428
IMT32929	0,52	0,54	0,45	0,71	0,50	0,47	0,49	0,45	0,75	0,75	0,72	0,83	0,5969
IMT34478	0,76	0,87	0,73	0,84	0,96	0,87	0,82	0,66	1,11	0,94	0,84	0,66	0,8366
IMT34479	0,28	0,34	0,48	0,42	0,30	0,30	0,27	0,26	0,23	0,26	0,23	0,22	0,2982
IMT34480	0,23	0,27	0,26	0,21	0,32	0,37	0,37	0,23	0,33	0,34	0,27	0,39	0,2978
IMT34485	0,15	0,09	0,08	0,09	0,11	0,09	0,09	0,10	0,10	0,09	0,09	0,08	0,0970
IMT34488	0,26	0,30	0,25	0,28	0,21	0,23	0,18	0,19	0,17	0,21	0,17	0,20	0,2181
IMT34489	0,25	0,23	0,25	0,24	0,18	0,19	0,23	0,24	0,22	0,24	0,23	0,19	0,2238
IMT34491	0,18	0,20	0,19	0,20	0,19	0,21	0,19	0,17	0,16	0,20	0,17	0,20	0,1893
IMT36943	0,19	0,28	0,24	0,24	0,16	0,21	0,20	0,19	0,16	0,22	0,22	0,20	0,2068
IMT36945	0,17	0,18	0,18	0,20	0,17	0,21	0,17	0,21	0,18	0,19	0,18	0,20	0,1857
IMT36946	0,19	0,12	0,14	0,10	0,13	0,11	0,11	0,11	0,14	0,08	0,11	0,11	0,1216
IMT36947	0,46	0,36	0,40	0,38	0,42	0,46	0,42	0,53	0,19	0,26	0,23	0,24	0,3621
IMT36948	0,21	0,23	0,23	0,39	0,42	0,53	0,37	0,46	0,24	0,22	0,23	0,25	0,3142
IMT36950	0,16	0,17	0,25	0,23	0,13	0,13	0,16	0,16	0,14	0,16	0,14	0,21	0,1693
IMT36952	1,00	1,19	1,18	1,08	1,08	1,26	1,40	1,19	1,35	1,56	1,57	1,21	1,2555
IMT38113	0,10	0,10	0,09	0,10	0,09	0,09	0,09	0,09	0,12	0,09	0,09	0,12	0,0973
IMT38115	0,20	0,17	0,18	0,25	0,16	0,25	0,22	0,24	0,18	0,36	0,35	0,33	0,2407
IMT38116	0,08	0,11	0,09	0,11	0,13	0,12	0,10	0,14	0,10	0,09	0,10	0,13	0,1093
IMT38119	0,20	0,20	0,22	0,26	0,18	0,19	0,18	0,19	0,29	0,33	0,43	0,32	0,2473
IMT39816	0,18	0,23	0,24	0,28	0,23	0,25	0,22	0,22	0,23	0,23	0,35	0,36	0,2503
IMT39819	1,77	1,49	1,67	1,75	2,37	2,46	2,36	2,37	1,99	1,46	2,39	1,71	1,9811

IMT39820	0,15	0,18	0,17	0,14	0,11	0,15	0,15	0,14	0,20	0,19	0,18	0,20	0,1636
IMT39824	0,26	0,27	0,30	0,28	0,14	0,16	0,15	0,19	0,15	0,14	0,14	0,15	0,1932
IMT39825	0,10	0,09	0,08	0,09	0,15	0,14	0,11	0,11	0,10	0,11	0,12	0,11	0,1089
IMT40504	0,25	0,35	0,25	0,29	0,19	0,20	0,21	0,20	0,30	0,32	0,35	0,39	0,2730
IMT40506	0,62	0,64	0,66	0,64	0,65	0,65	0,44	0,38	0,34	0,39	0,27	0,34	0,5013
IMT40507	0,14	0,22	0,17	0,18	0,14	0,14	0,11	0,14	0,08	0,09	0,12	0,11	0,1363
IMT41554	0,27	0,45	0,39	0,29	0,26	0,20	0,21	0,18	0,26	0,35	0,42	0,20	0,2898
RKI5962	0,25	0,30	0,29	0,26	0,21	0,25	0,22	0,32	0,30	0,23	0,30	0,25	0,2652
RKI5963	0,24	0,20	0,22	0,22	0,16	0,18	0,20	0,20	0,16	0,17	0,23	0,24	0,2011
RKI5964	0,22	0,20	0,19	0,22	0,17	0,11	0,11	0,11	0,20	0,19	0,13	0,21	0,1713
RKI5965	0,40	0,26	0,34	0,22	0,41	0,39	0,30	0,28	0,34	0,44	0,39	0,39	0,3448
RKI5966	0,18	0,21	0,24	0,15	0,14	0,26	0,14	0,16	0,14	0,26	0,14	0,16	0,1828
RKI5967	0,15	0,18	0,17	0,17	0,17	0,17	0,13	0,16	0,17	0,17	0,14	0,16	0,1614
RKI5968	0,48	0,66	0,64	0,66	0,39	0,41	0,54	0,52	0,39	0,41	0,53	0,52	0,5127
RKI5969	0,14	0,14	0,15	0,15	0,19	0,17	0,17	0,18	0,19	0,17	0,17	0,18	0,1666
RKI5970	0,20	0,17	0,18	0,17	0,18	0,18	0,18	0,18	0,18	0,19	0,18	0,18	0,1804
RKI5971	0,14	0,15	0,14	0,17	0,16	0,17	0,17	0,16	0,16	0,18	0,18	0,17	0,1632
RKI5972	0,10	0,09	0,11	0,09	0,09	0,10	0,10	0,09	0,09	0,10	0,09	0,09	0,0960
RKI5973	2,30	2,16	2,21	2,06	1,96	2,39	1,98	2,16	1,96	2,39	1,98	2,16	2,1418
RP62A	4,08	4,02	2,69	2,05	2,02	2,27	2,03	2,02	2,03	4,33	3,92	3,91	
	3,94	2,78	3,40	3,54	3,39	3,27	4,30	4,28	4,17	4,04	4,80	4,80	
	3,90	4,10			3,66	3,74			3,81	3,95			3,5071
TM300	0,10	0,13	0,06	0,08	0,07	0,05	0,08	0,08	0,05	0,05	0,07	0,09	
	0,08	0,07	0,09	0,08	0,07	0,05	0,10	0,12	0,18	0,11	0,11	0,13	
	0,14	0,15			0,13	0,09			0,12	0,09			0,0937

Measured optical density OD492 of all tested samples (three biological replicates). *S. epidermidis* RP62A and *S. carnosus* TM300 were used as positive and negative controls, respectively. A sample will be classified as biofilm positive if its measured optical density is at least three times the optical density of the negative control TM300.
